# Supplementary material for: Structural insight into the bulge-containing KRAS oncogene promoter G-quadruplex bound to berberine and coptisine
Source: Nat Commun. 2022 Oct 12;13:6016. doi: 10.1038/s41467-022-33761-4 (PMC9556435; doi:10.1038/s41467-022-33761-4)
Supplement: Supplementary file 1 — Supplementary information [file 41467_2022_33761_MOESM1_ESM.pdf]

Supporting Information

Structural Insight into the Bulge-containing *KRAS* Oncogene Promoter G-Quadruplex Bound to Berberine and Coptisine

Kai-Bo Wang,[1,3]* Yushuang Liu,[1,3] Jinzhu Li,[1] Chengmei Xiao,[1] Yingying Wang,[1] Wei Gu,[2] Yipu Li,[1] Yuan-Zheng Xia,[1] Tingdong Yan,[2] Ming-Hua Yang,[1] and Ling-Yi Kong[1]*

1. Jiangsu Key Laboratory of Bioactive Natural Product Research and State Key Laboratory of Natural Medicines, Department of Natural Medicinal Chemistry, China Pharmaceutical University, Nanjing 210009, China

2. School of Life Sciences, Shanghai University, Shanghai 200444, China

3. These authors contributed equally to this work.

Correspondence and requests for materials should be addressed to K.W. (email: kbwang@cpu.edu.cn) or L.K. (email: cpu_lykong@126.com)

Supplementary Table 1 The G4 DNA sequences used in this study.

**Sequence Name**	**DNA Sequence** (5′ to 3′)
KRAS-G4 (Pu24m1)	TGAGGGCGGTGTGGGAATAGGGAA
MYC-G4	TGAGGGTGGGTAGGGTGGGTAA
VEGF-G4	CAGGGCGGGCCTTGGGCGGGAT
Tel-hybrid1-G4	AAAGGGTTAGGGTTAGGGTTAGGGAA
Tel-hybrid2-G4	TTAGGGTTAGGGTTAGGGTTAGGGTT

Supplementary Table 2 ^{1}H and C6/C8 ^{13}C chemical shifts (ppm) of Pu24m1 DNA at 25 °C in pH 7, 50 mM K^{+}-containing solution.

Base	H1/H2/H5/Me	H6/H8	H1′	H2′, H2″	H3′	H4′	H5′, H5″*	C6/C8
**T1**	1.64	7.23	5.78	1.64, 2.06	4.43	3.81	3.48, 3.48	139.2
**G2**		7.42	5.67	2.61, 2.44	4.72	4.08	3.73, 3.76	139.3
**A3**	7.83	8.04	5.93	2.40, 2.59	4.85	3.96	3.64, 3.88	141.2
**G4**	11.79	8.07	6.09	2.80, 3.03	4.99	4.47	4.05, 4.14	137.8
**G5**	11.18	7.63	6.12	2.52, 2.88	4.97	4.55	4.23, 4.31	137.0
**G6**	11.25	7.70	6.30	2.80, 2.69	5.14	4.63	4.33, 4.40	137.8
**C7**	6.21	8.04	6.51	2.42, 2.75	5.10	4.64	4.28, 4.40	143.9
**G8**	11.82	8.03	6.17	2.35, 2.93	5.19	4.49	4.28, 4.35	137.6
**G9**	11.27	7.99	6.29	2.71, 2.87	5.19	4.47	4.28, 4.35	138.5
**T10**	1.77	7.42	5.76	2.17, 2.22	4.64	4.42	4.10, 4.34	139.4
**G11**	11.21	7.68	6.48	2.46, 3.07	5.02	4.69	4.17, 4.23	137.7
**T12**	1.99	8.00	6.58	2.55, 2.66	5.20	4.60	4.31, 4.45	140.1
**G13**	11.74	8.12	6.18	2.62, 2.99	5.13	4.51	4.30, 4.39	137.9
**G14**	11.39	7.93	6.02	2.62, 2.62	4.88	4.44	4.29, 4.29	138.5
**G15**	11.20	7.72	6.33	2.45, 2.50	4.74	3.92	3.04, 3.30	137.9
**A16**	8.05	8.28	6.30	2.83, 2.74	4.92	4.39	4.03, 4.14	141.9
**A17**	8.11	8.49	6.48	2.99, 2.85	5.12	4.55	4.15, 4.30	142.4
**T18**	1.92	7.57	6.09	1.70, 2.17	4.91	4.55	4.14, 4.30	139.1
**A19**	8.12	8.08	6.27	2.85, 2.80	5.12	4.42	3.98, 4.12	141.1
**G20**	11.37	8.08	6.02	2.91, 2.62	5.02	4.50	4.28, 4.28	137.9
**G21**	11.31	7.93	6.05	2.77, 2.75	5.10	4.58	4.26, 4.31	137.8
**G22**	10.80	7.62	6.02	2.50, 2.78	4.97	4.51	4.24, 4.32	137.0
**A23**	7.48	7.80	5.92	2.51, 2.35	4.84	4.31	4.07, 4.15	140.8
**A24**	7.50	7.94	6.02	2.42, 2.30	4.56	4.15	4.05, 4.10	141.3

Note: *Assignments are not stereospecific.

Supplementary Table 3 Inter-residue NOE cross-peaks of the 5′-end capping structure of Pu24m1 DNA.

	**G2**					**A3**			
**T1**	**H8**	**H3′**	**H4′**	**H5′**	**H5″**	**H8**	**H2**	**H2′**	**H2″**
**H1′**				W	W				
**H2′**	W					W			
**H2″**	W	W	VW			W			
**H3′**	M			M	M	W			
**H4′**	W								
**H6**								VW	W
**Me**							W		

	**A3**			**G4**	**G8**	**G13**
**G2**	**H8**	**H5′**	**H5″**	**H1**	**H1**	**H1**
**H1′**	W	W	W	M	M	
**H2′**	M			M		
**H2″**	M			M		
**H3′**	M					
**H4′**	W					
**H5′**	W					
**H5″**	W					
**H8**	M			W	W	W

	**G4**		**G20**	
**A3**	**H8**	**H1**	**H1**	**H1′**
**H1′**	M		M	
**H2′**	W			
**H2″**	W			
**H3′**	W			
**H4′**	M			
**H2**			VW	W
**H8**		W	M	

Note: M = medium intensity, W = weak intensity, VW = very weak intensity.

Supplementary Table 4 Inter-residue NOEs of the 3′-end capping structure of Pu24m1 DNA.

	**G22**							**G6**	
**A23**	**H1**	**H8**	**H1′**	**H2′**	**H2″**	**H3′**	**H4′**	**H1**	**H8**
**H8**		M	M	M	OL	W	W		
**H2**	M							W	M

	**A23**						
**A24**	**H8**	**H2**	**H1′**	**H2′**	**H2″**	**H3′**	**H4′**
**H8**	M		M	M	M	W	W
**H1′**		W					
**H5′**			W				
**H5″**			W				

	**G6**						**G11**	**A23**	**A24**
**T10**	**H1**	**H1′**	**H2′**	**H2″**	**H3′**	**H8**	**H8**	**H2**	**H2**
**Me**	VW	M	W	M	W	W		W	W
**H6**	W						W		
**H1′**	M						M		
**H3′**							W		
**H4′**							M		
**H5′**							W		
**H5″**							W		

Note: M = medium intensity, W = weak intensity, VW = very weak intensity, OL = overlapped.

Supplementary Table 5 Inter-residue NOEs of the A16-A19 4-nt loop structure of Pu24m1 DNA.

	**G15**				**A17**				
**A16**	**H3′**	**H4′**	**H5′**	**H5″**	**H8**	**H1′**	**H2′**	**H2″**	**H4′**
**H8**	W	M	M	M	M				
**H2**			W	W					
**H1′**		W			M	W	W	OL	W
**H2′**					M				
**H2″**					M				
**H3′**		W			W				
**H4′**		W			W				
**H5′**		M							
**H5″**		M							

	**T18**				
**A17**	**H6**	**Me**	**H2′**	**H2″**	**H3′**
**H8**	VW	W			
**H1′**	W	M	W	VW	W
**H2′**	W	W			
**H2″**	M	M			
**H3′**	M	W			

	**A19**				
**T18**	**H8**	**H3′**	**H4′**	**H5′**	**H5″**
**H6**	M				
**H1′**	W	W	W	M	M
**H2′**	M				
**H2″**	M				
**H3′**	W				

	**A16**	**A17**
**A19**	**H8**	**H8**
**H8**	M	M

Note: M = medium intensity, W = weak intensity, VW = very weak intensity, OL = overlapped.

Supplementary Table 6 ^{1}H and C6/C8 ^{13}C chemical shifts (ppm) of BER-Pu24m1 complex at 25 °C in pH 7, 50 mM K^{+}-containing solution.

Base	H1/H2/H5/Me	H6/H8	H1′	H2′, H2″	H3′	H4′	H5′, H5″*	C6/C8
**T1**	1.63	7.21	5.76	1.55, 1.97	4.39	3.77	3.43, 3.43	139.3
**G2**		7.49	5.58	2.20, 2.31	4.70	3.97	3.55, 3.68	138.7
**A3**	7.91	8.17	6.20	2.63, 2.75	4.95	4.26	3.97, 3.97	141.6
**G4**	11.52	8.09	6.06	2.75, 3.02	5.01	4.50	4.19, 4.19	138.2
**G5**	11.11	7.64	6.13	2.57, 2.91	5.00	4.55	4.30, 4.38	137.2
**G6**	10.90	7.71	6.20	2.79, 2.70	5.15	4.63	4.30, 4.38	137.7
**C7**	6.21	8.05	6.51	2.44, 2.76	5.09	4.60	4.30, 4.40	144.0
**G8**	11.43	8.00	6.13	2.34, 2.93	5.19	4.51	4.27, 4.36	137.7
**G9**	10.92	7.84	6.26	2.74, 2.73	5.20	4.48	4.30, 4.37	138.1
**T10**	1.94	7.60	6.21	2.45, 2.45	4.84	4.38	4.19, 4.27	139.8
**G11**	10.88	7.55	6.50	2.42, 2.99	5.07	4.73	4.19, 4.23	136.8
**T12**	2.00	8.01	6.57	2.56, 2.63	5.19	4.59	4.30, 4.42	140.2
**G13**	11.30	7.95	6.08	2.58, 2.88	5.01	4.48	4.31, 4.40	137.4
**G14**	11.18	7.75	5.97	2.51, 2.51	4.84	4.40	4.26, 4.26	137.9
**G15**	10.60	7.72	6.30	2.54, 2.59	4.76	3.90	2.94, 3.26	137.8
**A16**	8.03	8.29	6.30	2.76, 2.84	4.94	4.39	4.05, 4.16	142.0
**A17**	8.11	8.50	6.47	2.99, 2.83	5.12	4.54	4.13, 4.29	142.4
**T18**	1.93	7.57	6.09	1.66, 2.13	4.81	4.25	3.98, 4.14	139.1
**A19**	8.11	8.11	6.30	2.84, 2.84	5.13	4.42	3.98, 4.33	141.1
**G20**	11.42	7.99	6.01	2.58, 2.88	5.01	4.45	4.25, 4.26	137.8
**G21**	11.19	7.82	6.04	2.71, 2.69	5.08	4.56	4.25, 4.33	137.7
**G22**	10.80	7.50	6.13	2.29, 2.49	5.03	4.49	4.24, 4.29	136.9
**A23**	7.55	8.20	6.07	2.61, 2.57	4.95	4.40	4.11, 4.17	141.7
**A24**	7.55	8.11	5.99	2.29, 2.46	4.82	4.59	4.06, 4.06	141.7

Note: *Assignments are not stereospecific.

Supplementary Table 7 Intermolecular NOEs between BER and Pu24m1 DNA.

5′-end BER

	**G4**	**G8**			**G13**			**G20**	**A3**
**BER**	**H1**	**H1**	**H8**	**H1′**	**H1**	**H1′**	**H8**	**H1**	**H2**
**H8**	VW	W			W			W	M
**H4**	W								
**H51**									M
**H52**									M
**H61**	M	M			M			M	M
**H62**	M	M			M			M	M
**HA**						W			W
**HB**						M	M		
**HC1**			M	W					
**HC2**			M	W					

3′-end BER

	**G6**		**G11**		**G15**		**G22**	**A23**	**A24**
**Ber**	**H1**	**H8**	**H1**	**H8**	**H1**	**H8**	**H1**	**H2**	**H2**
**H8**	W		W		VW		VW		
**H4**							W		
**H51**								M	
**H52**								M	
**H61**	M		M		M		M	M	
**H62**	M		M		M		M	M	
**HA**						W		OL	OL
**HB**				W					
**HC1**		M							
**HC2**		M							

Note: M = medium intensity, W = weak intensity, VW = very weak intensity, OL = overlapped.

Supplementary Table 8 Inter-residue NOE cross-peaks of the 5′-end capping structure of the BER-Pu24m1 complex.

	**G2**			
**T1**	**H8**	**H4′**	**H5′**	**H5″**
**H1′**	W	W	M	M
**H2′**	M			
**H2″**	M			
**H3′**	M			
**H4′**	W			
**H6**	M			

	**A3**		
**G2**	**H8**	**H2**	**H1′**
**H1′**	W	W	W
**H2′**	M		
**H2″**	M		
**H3′**	M		
**H8**	M		

	**G4**		**G13**	**G20**	
**A3**	**H8**	**H1**	**H1**	**H1**	**H1′**
**H1′**	M				
**H2′**	M				
**H2″**	OL				
**H3′**	W				
**H4′**	W				
**H5′**	VW				
**H5″**	VW				
**H2**		W	W	M	
**H8**					VW

Note: M = medium intensity, W = weak intensity, VW = very weak intensity, OL = overlapped.

Supplementary Table 9 Inter-residue NOEs of the 3′-end capping structure of the BER-Pu24m1 complex.

	**G11**	**G15**	**G22**						
**A23**	**H1**	**H1**	**H1**	**H8**	**H1′**	**H2′**	**H2″**	**H3′**	**H4′**
**H8**				M	W	M	M	W	VW
**H2**	W	W	W						
**H3′**					W				
**H4′**					OL				
**H5′**					M				
**H5″**					M				

	**A23**					
**A24**	**H8**	**H1′**	**H2′**	**H2″**	**H3′**	**H4′**
**H8**	M	M	M	M	W	W
**H5′**		W				
**H5″**		W				

	**G6**		**G9**					**G11**
**T10**	**H8**	**H1′**	**H8**	**H1′**	**H2′**	**H2″**	**H3′**	**H8**
**H1′**								W
**H3′**								W
**H4′**								M
**H5′**			W	W				
**H5″**			W	W				
**H6**					W	W	VW	
**Me**	VW	M						

Note: M = medium intensity, W = weak intensity, VW = very weak intensity, OL = overlapped.

Supplementary Table 10 Inter-residue NOEs of the A16-A19 4-nt loop structure of BER-Pu24m1 complex.

	**G15**				**G14**			**A17**				
**A16**	**H3′**	**H4′**	**H5′**	**H5″**	**H4′**	**H5′**	**H5″**	**H8**	**H1′**	**H2′**	**H2″**	**H4′**
**H8**	W	M	M	M				M				
**H2**		W	W	W	M	W	W					
**H1′**								M	W	W	OL	W
**H2′**								M				
**H2″**								M				
**H3′**		W						W				
**H4′**		W						W				
**H5′**		M										
**H5″**		M										

	**T18**				
**A17**	**H6**	**Me**	**H2′**	**H2″**	**H3′**
**H8**	VW	W			
**H1′**	W	M	W	VW	W
**H2′**	W				
**H2″**	M				
**H3′**	M				
**H4′**	W				

	**A19**					
**T18**	**H8**	**H1′**	**H3′**	**H4′**	**H5′**	**H5″**
**H6**	M					
**H1′**	W		W	W	M	M
**H2′**	M	VW				
**H2″**	M	W				
**H3′**	M					

	**A16**	**A17**			
**A19**	**H8**	**H8**	**H1′**	**H2′**	**H2″**
**H8**	M	M	VW	W	OL

Note: M = medium intensity, W = weak intensity, VW = very weak intensity, OL = overlapped

Supplementary Table 11 ^{1}H and C6/C8 ^{13}C chemical shifts (ppm) of COP-Pu24m1 complex at 25 °C in pH 7, 50 mM K^+-containing solution.

Base	H1/H2/H5/Me	H6/H8	H1′	H2′, H2″	H3′	H4′	H5′, H5″*	C6/C8
**T1**	1.62	7.22	5.74	1.57, 2.01	4.42	3.78	3.46, 3.46	139.3
**G2**		7.52	5.58	2.21, 2.28	4.69	3.98	3.52, 3.67	138.8
**A3**	7.91	8.20	6.24	2.67, 2.76	4.96	4.22	3.99, 3.99	141.7
**G4**	11.52	8.09	6.06	2.75, 3.01	5.01	4.50	4.19, 4.30	138.2
**G5**	11.06	7.65	6.12	2.57, 2.91	5.00	4.55	4.30, 4.30	137.2
**G6**	10.96	7.75	6.32	2.78, 2.71	5.14	4.63	4.30, 4.39	138
**C7**	6.21	8.04	6.51	2.43, 2.76	5.10	4.61	4.30, 4.40	144
**G8**	11.39	7.99	6.13	2.34, 2.94	5.20	4.52	4.51, 4.51	137.7
**G9**	10.95	7.87	6.25	2.75, 2.75	5.20	4.49	4.32, 4.36	138.2
**T10**	1.96	7.60	6.17	2.38, 2.43	4.92	4.36	4.19, 4.28	139.8
**G11**	10.85	7.62	6.51	2.46, 3.02	5.05	4.73	4.21, 4.24	137.2
**T12**	2.00	8.01	6.57	2.56, 2.64	5.20	4.59	4.30, 4.41	140.2
**G13**	11.31	8.01	6.10	2.55, 2.92	5.05	4.50	4.51, 4.51	137.8
**G14**	11.15	7.77	5.96	2.53, 2.53	4.85	4.41	4.28, 4.28	138.1
**G15**	10.48	7.72	6.30	2.51, 2.56	4.77	3.85	3.01, 3.32	137.9
**A16**	8.04	8.30	6.31	2.77, 2.83	4.93	4.40	4.06, 4.16	142
**A17**	8.06	8.49	6.50	2.98, 2.84	5.12	4.54	4.15, 4.15	142.3
**T18**	1.94	7.55	6.09	1.72, 2.16	4.84	4.28	4.15, 4.15	139.1
**A19**	8.14	8.15	6.30	2.81, 2.81	5.11	4.43	4.00, 4.14	141.1
**G20**	11.40	8.01	5.97	2.90, 2.62	5.01	4.48	4.22, 4.27	137.7
**G21**	11.15	7.84	6.03	2.72, 2.72	5.10	4.56	4.26, 4.32	137.8
**G22**	10.66	7.50	6.13	2.23, 2.39	5.04	4.49	4.26, 4.30	137
**A23**	7.47	8.23	6.06	2.61, 2.64	4.97	4.42	4.08, 4.17	141.7
**A24**	7.60	8.15	6.05	2.50, 2.33	4.62	4.29	4.09, 4.09	141.8

Note: *Assignments are not stereospecific.

Supplementary Table 12 Intermolecular NOEs between COP and Pu24m1 DNA.

5′-end COP

	**G4**	**G8**			**G13**		**G20**		**A3**
**COP**	**H1**	**H1**	**H8**	**H1′**	**H1**	**H8**	**H1**	**H8**	**H2**
**H8**	VW	W			W		W		M
**H4**	W	W							
**H51**									M
**H52**									M
**H61**	M	M			M		M		M
**H62**	M	M			M		M		M
**H11**						W			
**H12**						W			
**HA**			M	M					
**HC**								OL	

3′-end COP

	**G6**		**G11**	**G15**		**G22**	**A23**	**A24**
**COP**	**H1**	**H8**	**H1**	**H1**	**H8**	**H1**	**H2**	**H1′**
**H8**	W		W	VW		VW	W	W
**H51**							M	
**H52**							M	
**H61**	M		M	M		M	M	
**H62**	M		M	M		M	M	
**HA**		M						
**HC**					M			

Note: M = medium intensity, W = weak intensity, VW = very weak intensity, OL = overlapped.

Supplementary Table 13 Inter-residue NOE cross-peaks of the 5′-end capping structure of COP-Pu24m1 complex.

	**G2**				
**T1**	**H8**	**H3′**	**H4′**	**H5′**	**H5″**
**H1′**	W	VW	W	M	M
**H2′**	M				
**H2″**	M				
**H3′**	M				
**H4′**	W				
**H6**	M				

	**A3**		
**G2**	**H8**	**H2**	**H1′**
**H1′**	W	W	W
**H2′**	M		
**H2″**	M		
**H3′**	M		
**H4′**	W		
**H8**	W		

	**G4**		**G13**	**G20**	
**A3**	**H8**	**H1**	**H1**	**H1**	**H1′**
**H1′**	M				
**H2′**	M				
**H2″**	OL				
**H3′**	W				
**H4′**	W				
**H5′**	VW				
**H5″**	VW				
**H2**		W	W	M	
**H8**					VW

Note: M = medium intensity, W = weak intensity, VW = very weak intensity, OL = overlapped.

Supplementary Table 14 Inter-residue NOEs of the 3′-end capping structure of COP-Pu24m1 complex.

	G15	G22						
**A23**	**H1**	**H1**	**H8**	**H1′**	**H2′**	**H2″**	**H3′**	**H4′**
**H8**			W	W	M	M	W	VW
**H2**	W	W						
**H3′**				W				
**H4′**				W				
**H5′**				M				
**H5″**				M				

	A23					
**A24**	**H8**	**H1′**	**H2′**	**H2″**	**H3′**	**H4′**
**H8**	M	M	M	M	W	W

	G6						G9		G11
**T10**	**H8**	**H1′**	**H2′**	**H2″**	**H3′**	**H4′**	**H8**	**H1′**	**H8**
**H1′**									W
**H3′**									W
**H4′**									M
**H5′**							W	W	
**H5″**							W	W	
**H6**									
**Me**	VW	M	M	M	W	W			

Note: M = medium intensity, W = weak intensity, VW = very weak intensity.

Supplementary Table 15 Inter-residue NOEs of the A16-A19 4-nt loop structure of COP-Pu24m1 complex.

	**G15**				**G14**	**A17**				
**A16**	**H3′**	**H4′**	**H5′**	**H5″**	**H4′**	**H8**	**H1′**	**H2′**	**H2″**	**H4′**
**H8**	W	M	M	M		M				
**H2**		W	W	W	M					
**H1′**						M	W	W	OL	W
**H2′**						M				
**H2″**						OL				
**H3′**		W				W				
**H4′**		W				W				
**H5′**		M	M	M						
**H5″**		M	M	M						

	**T18**				
**A17**	**H6**	**Me**	**H2′**	**H2″**	**H3′**
**H8**	VW	W			
**H1′**	W	M	W	VW	W
**H2′**	W				
**H2″**	M				
**H3′**	M				
**H4′**	W				

	**A19**					
**T18**	**H8**	**H1′**	**H3′**	**H4′**	**H5′**	**H5″**
**H6**	M					
**H1′**	W		W	W	M	M
**H2′**	M	VW				
**H2″**	M	W				
**H3′**	M					

	**A16**	**A17**
**A19**	**H8**	**H8**
**H8**	M	M

Note: M = medium intensity, W = weak intensity, VW = very weak intensity, OL = overlapped.

Supplementary Table 16 Proton chemical shifts of free-ligands and bound-ligands at 25 °C in pH 7, 50 mM K^+-containing solution.

**Proton**	**Free-BER**	**Bound-BER**	**Free-COP**	**Bound-COP**
**H51**	3.26	2.72	3.26	2.67
**H52**	3.26	2.79	3.26	2.74
**H61**	4.85	4.60	4.84	4.63
**H62**	4.85	4.60	4.84	4.63
**HA**	4.13	3.76	6.13	5.78
**HB**	4.14	3.86	-	-
**HC**	6.13	5.82	6.46	6.12
**H1**	7.55	6.66	7.57	6.55
**H4**	6.99	6.12	7.00	6.13
**H8**	9.67	9.05	9.62	8.96
**H11**	8.08	7.50	7.86	7.24
**H12**	7.99	7.27	7.82	7.09
**H13**	8.55	7.84	8.59	7.86

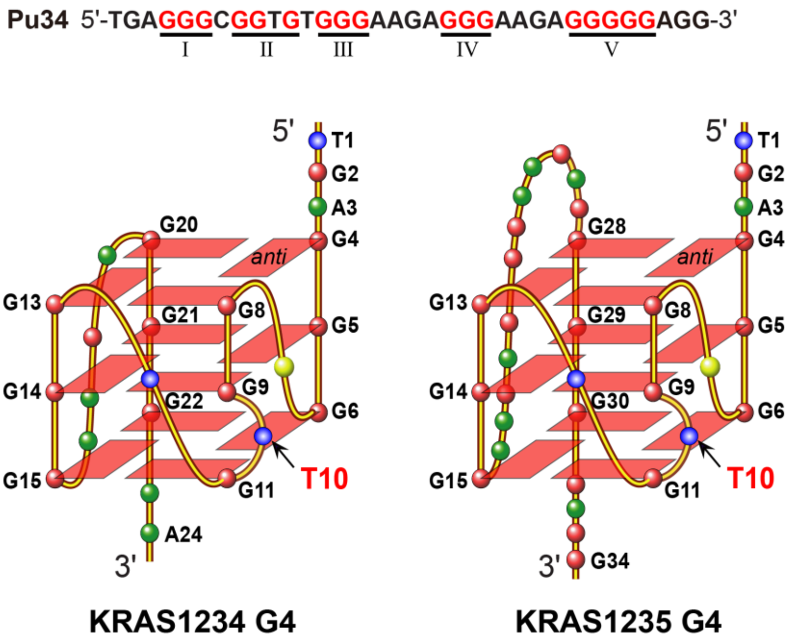

Supplementary Fig. 1 Schematic of KRAS1234 and KRAS1235 G4s in the human *KRAS* oncogene promoter.

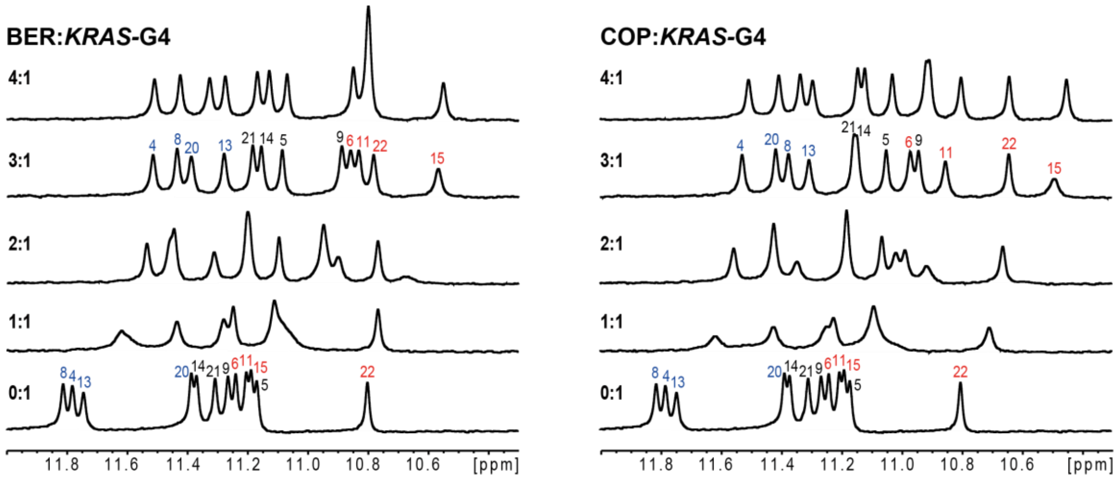

Supplementary Fig. 2 1D ^{1}H NMR titration of Pu24m1 DNA with berberine (BER) and coptisine (COP), respectively. Conditions: 150 μM DNA, pH 7, 50 mM K^+ solution, 25 °C, without DMSO-d_6 (compound stock was prepared with 50 mM K^+ solution).

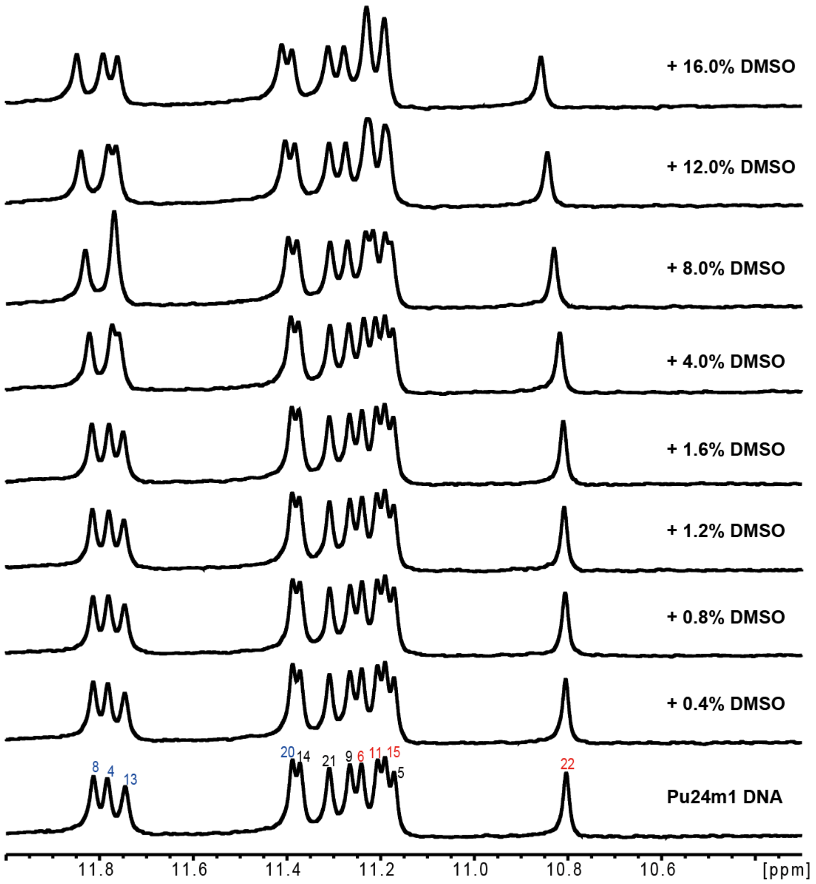

Supplementary Fig. 3 1D ^{1}H NMR titration of Pu24m1 DNA with increasing concentration of DMSO-d_6. Volume percentages of DMSO-d_6 are shown on the right side of the spectra. Conditions: 150 μM DNA, pH 7, 50 mM K^+ solution, 25 °C.

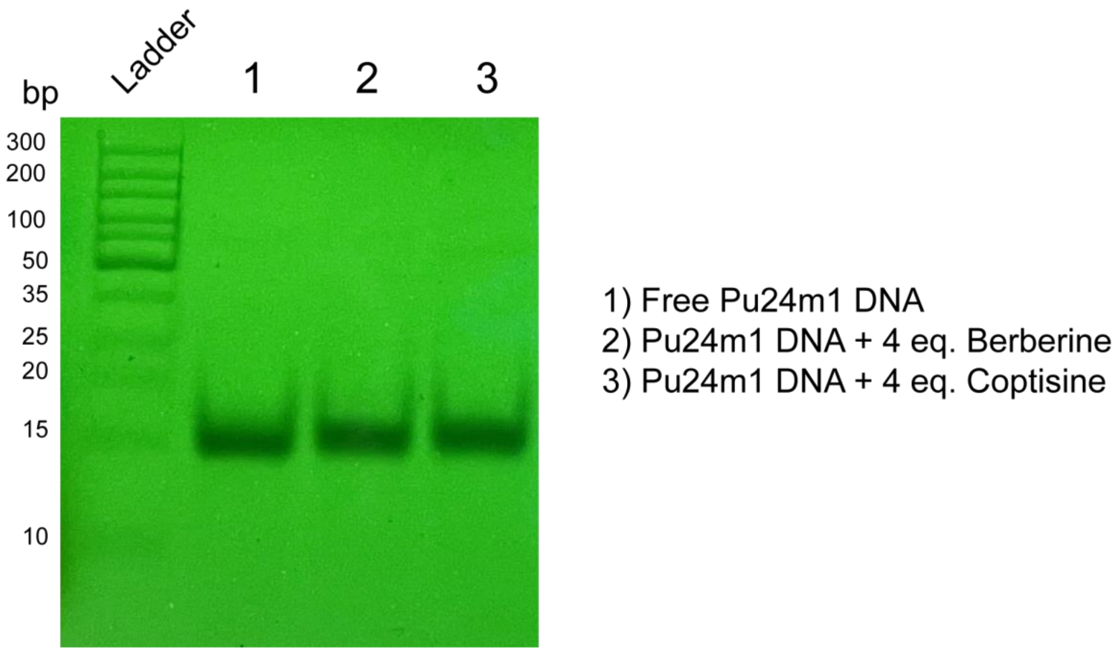

Supplementary Fig. 4 Native EMSA gel of Pu24m1 DNA and its complex with berberine and coptisine, respectively. Conditions: 150 μM DNA, pH 7, 50 mM K^+ solution. Each sample contained 5 μL of 150 μM DNA. DNA bands were visualized using UV light at 260 nm. The experiment was run in duplicate.

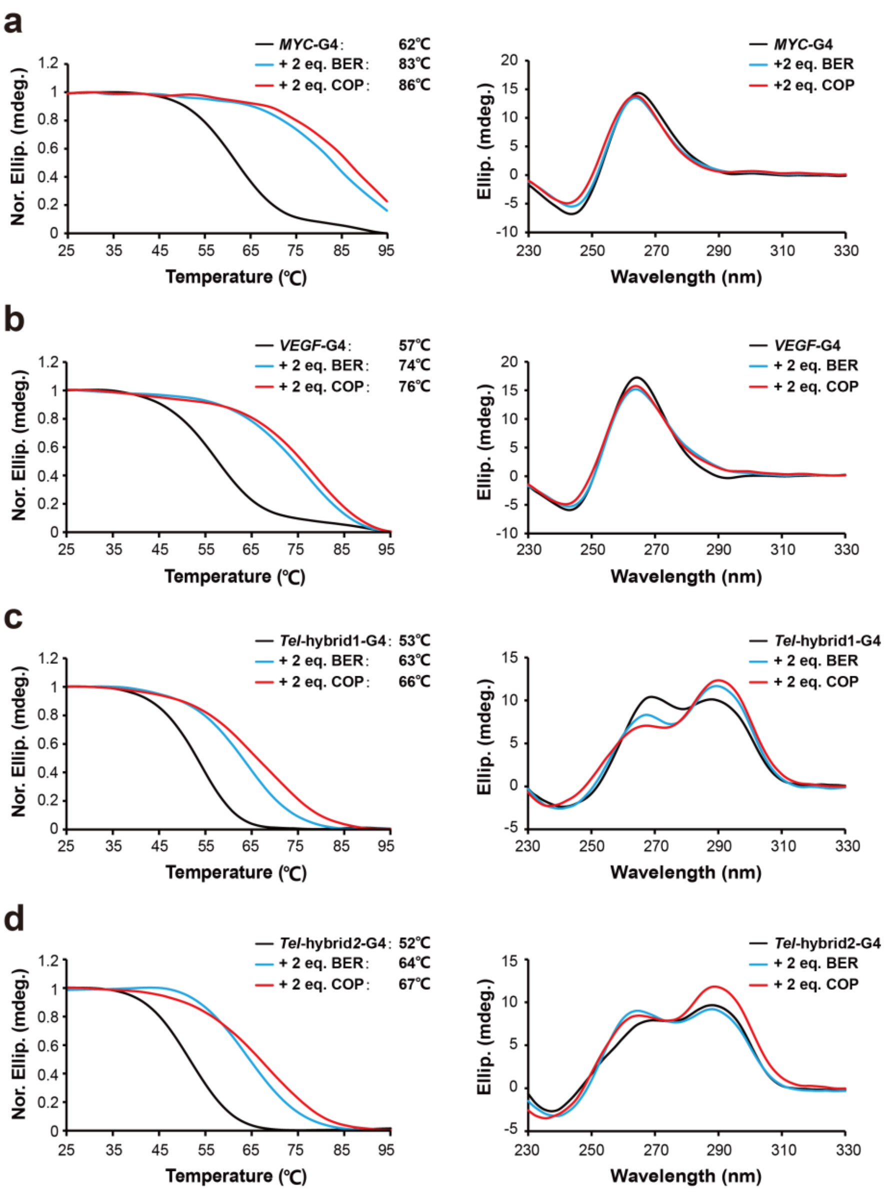

Supplementary Fig. 5 CD thermal melting curves and CD spectra of *MYC*-G4 (a), *VEGF*-G4 (b), *Tel*-hybrid1-G4 (c), and *Tel*-hybrid2-G4 (d) with berberine and coptisine, respectively. Conditions: 20 μM DNA, 40 μM compound, pH 7, 0.5-50 mM K^+ solution. The melting temperature (T_m) was obtained at the intersection between the median of the fitted baselines and the melting curve, which is shown at top of each left figure.

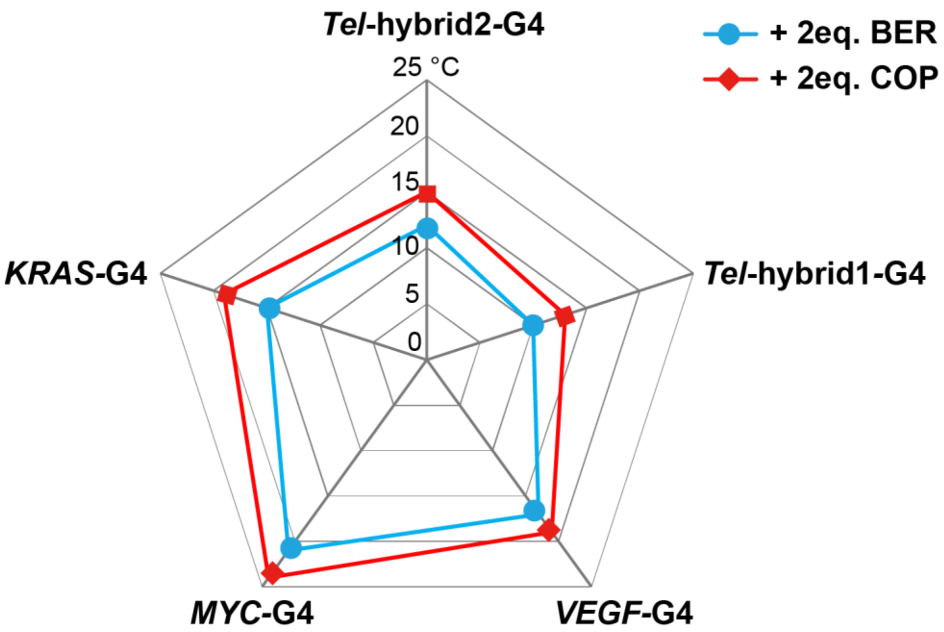

Supplementary Fig. 6 Structural selectivity profile results were shown by the ΔT_m values obtained from CD melting experiments. Details of the DNA sequences of G4s are provided in Supplementary Table S1.

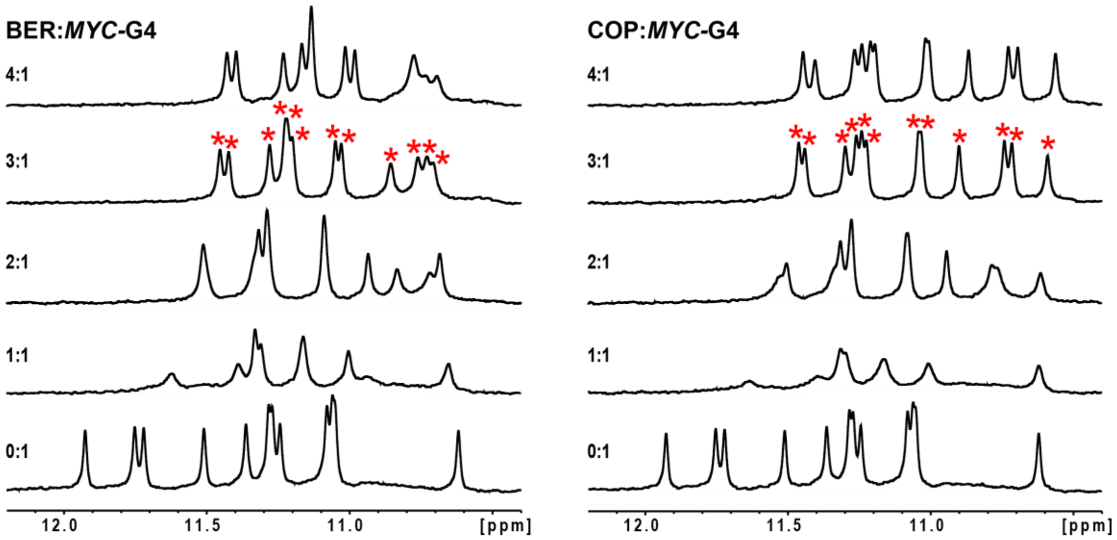

Supplementary Fig. 7 1D ^{1}H NMR titration of *MYC*-G4 DNA with berberine (BER) and coptisine (COP), respectively. New emerged 12 imino peaks are marked with red asterisks. Conditions: 150 μM DNA, pH 7, 50 mM K^+ solution, 25 °C, DMSO-d_6 < 1.5%.

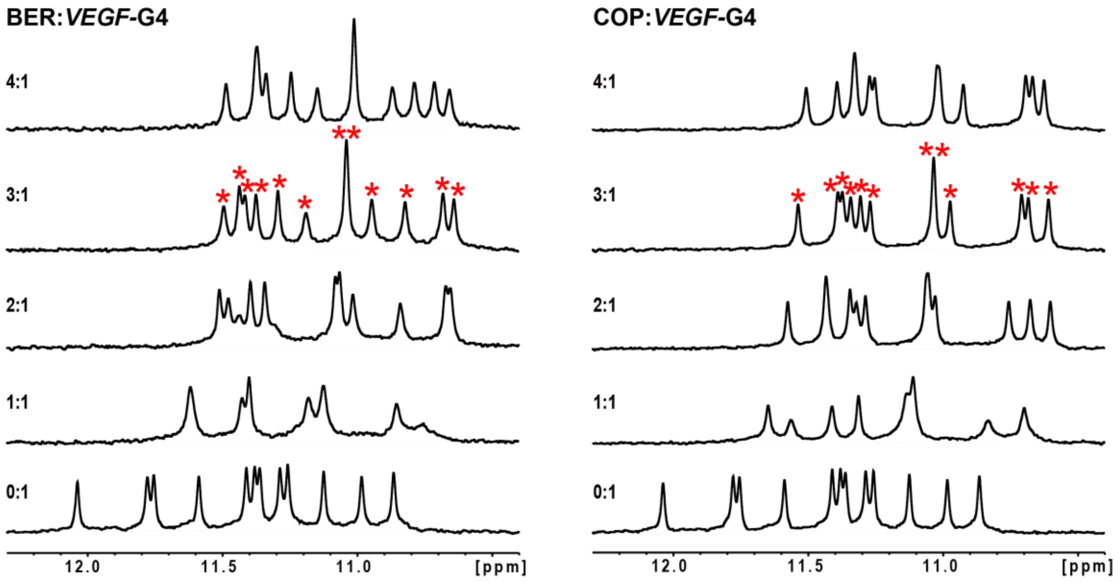

Supplementary Fig. 8 1D ^{1}H NMR titration of *VEGF*-G4 DNA with berberine (BER) and coptisine (COP), respectively. New emerged 12 imino peaks are marked with red asterisks. Conditions: 150 μM DNA, pH 7, 50 mM K^+ solution, 25 °C, DMSO-d_6 < 1.5%.

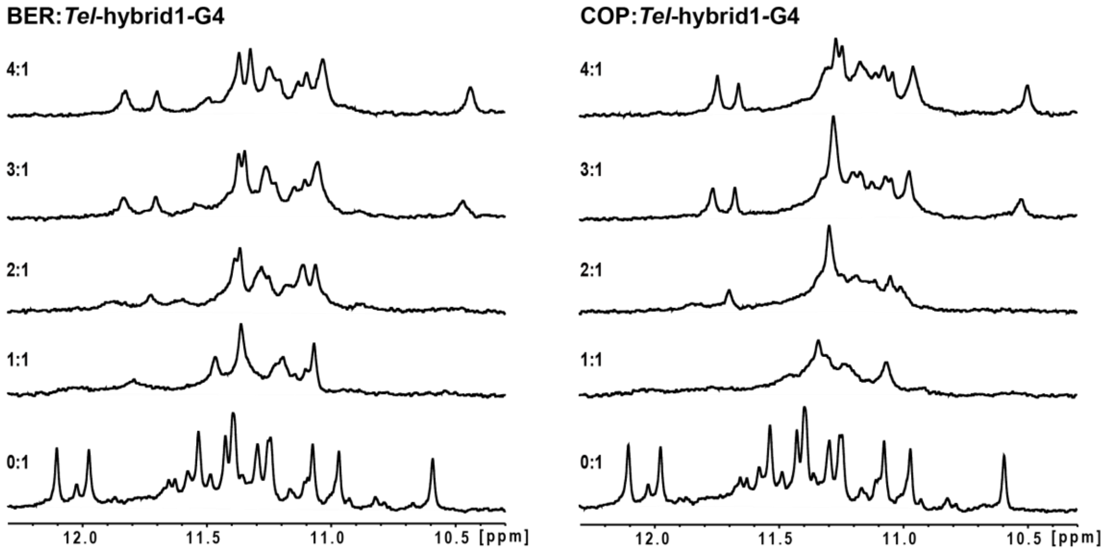

Supplementary Fig. 9 1D ^{1}H NMR titration of *Tel*-hybid1-G4 DNA with berberine (BER) and coptisine (COP), respectively. Conditions: 150 μM DNA, pH 7, 50 mM K^{+} solution, 25 °C, DMSO-d_6 < 1.5%.

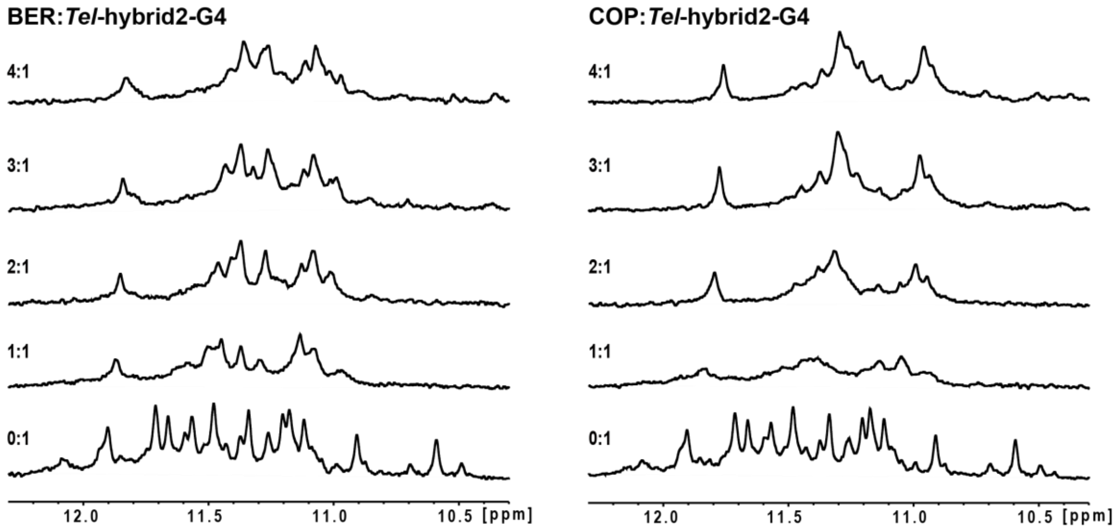

Supplementary Fig. 10 1D ^{1}H NMR titration of *Tel*-hybid2-G4 DNA with berberine (BER) and coptisine (COP), respectively. Conditions: 150 μM DNA, pH 7, 50 mM K^{+} solution, 25 °C, DMSO-d_6 < 1.5%.

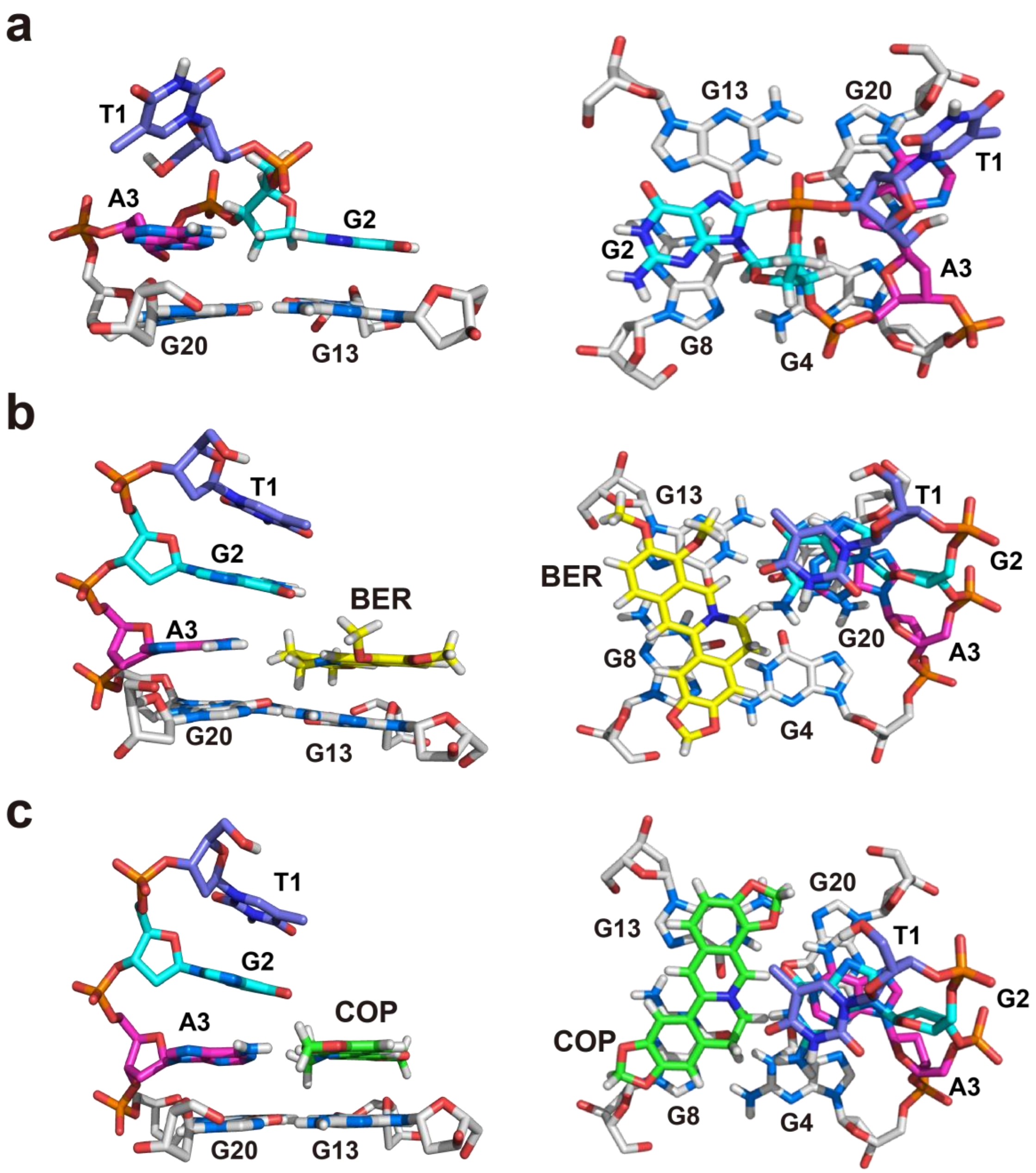

Supplementary Fig. 11 Side (left) and top (right) view of the 5′-end capping structure of (a) Pu21m1 DNA G4, (b) BER-Pu21m1 complex, and (c) COP-Pu24m1 complex. Gray, tetrad guanine; cyan, loop guanine (G2); magenta, adenine; marine, thymine; yellow, BER; and green, COP.

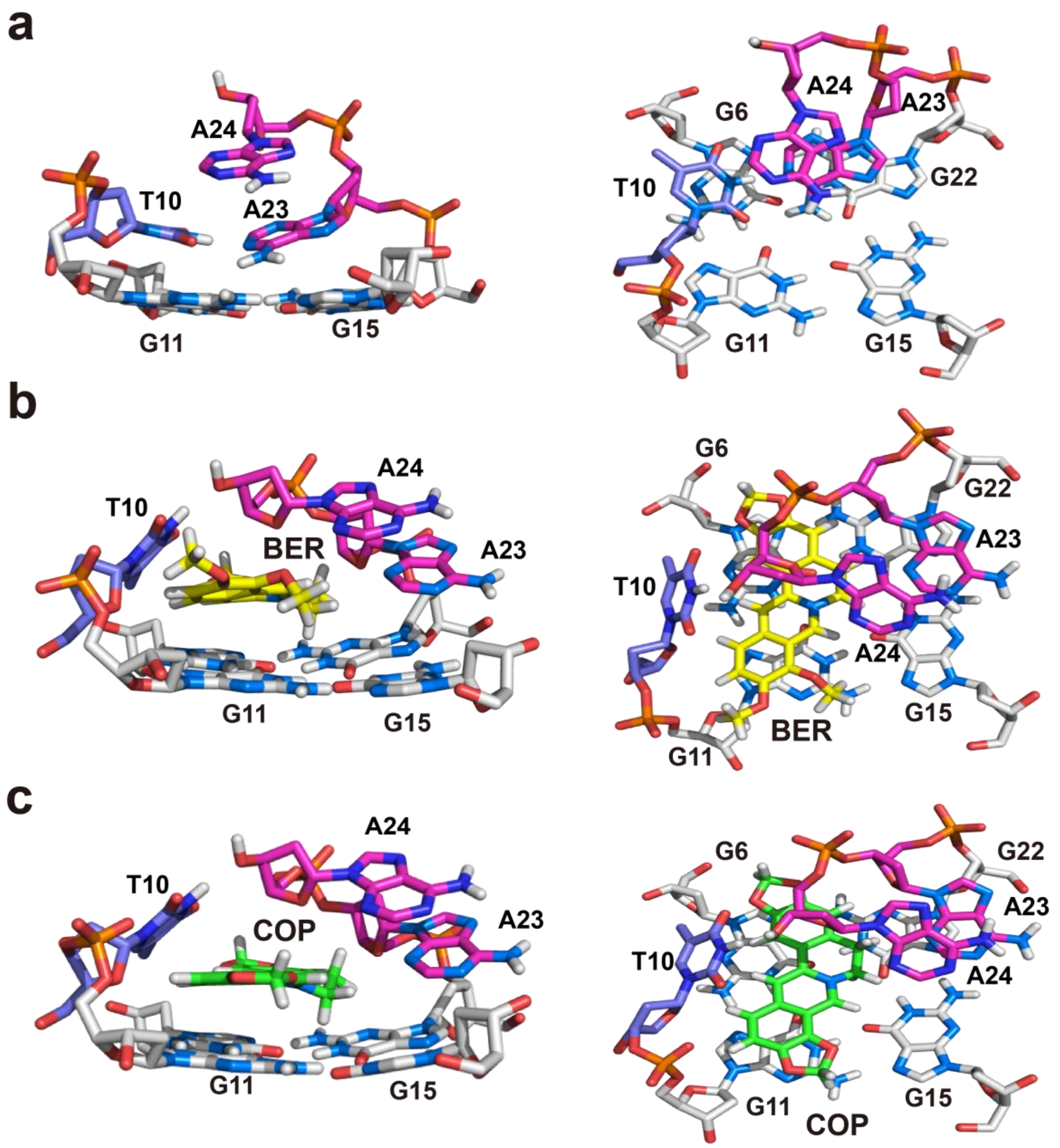

Supplementary Fig. 12 Side (left) and top (right) view of the 3′-end capping structure of (a) Pu21m1 DNA G4, (b) BER-Pu21m1 complex, and (c) COP-Pu24m1 complex. Gray, guanine; magenta, adenine; marine, thymine; yellow, BER; and green, COP.

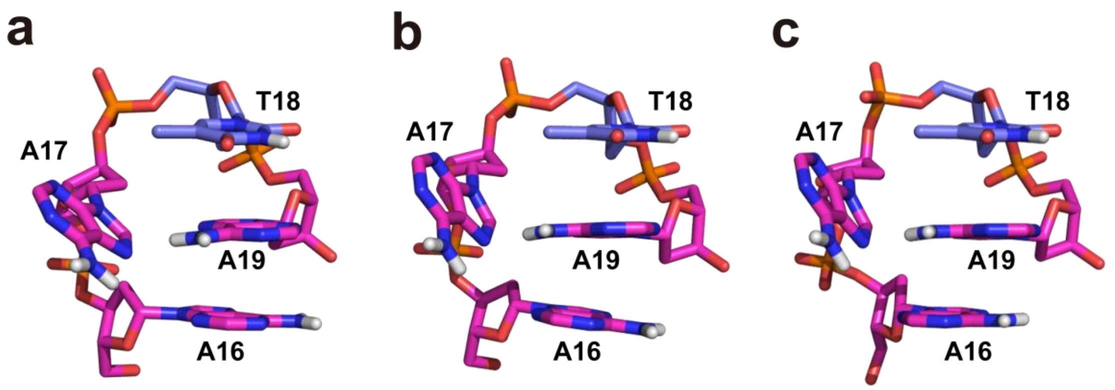

Supplementary Fig. 13 Side view of the 4-nt propeller loop structure of (a) Pu21m1 DNA, (b) BER-Pu21m1 complex, and (c) COP-Pu24m1 complex. Magenta, adenine; marine, thymine.

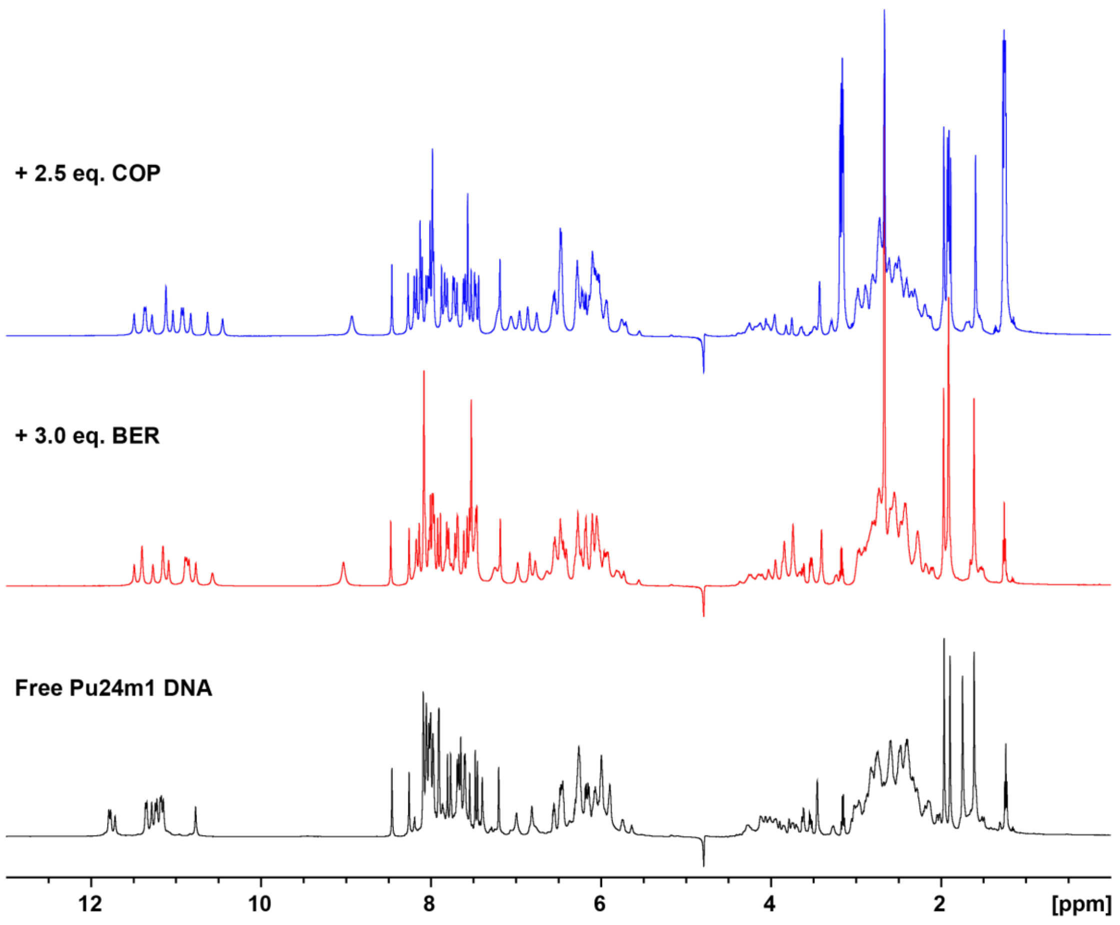

Supplementary Fig. 14 The full 1D ^{1}H NMR spectra of Pu24m1 DNA with and without berberine (BER) and coptisine (COP), respectively. Conditions: 1.5 mM DNA, pH 7, 50 mM K^+ solution, 25 °C, DMSO-d_6 < 3.5%.

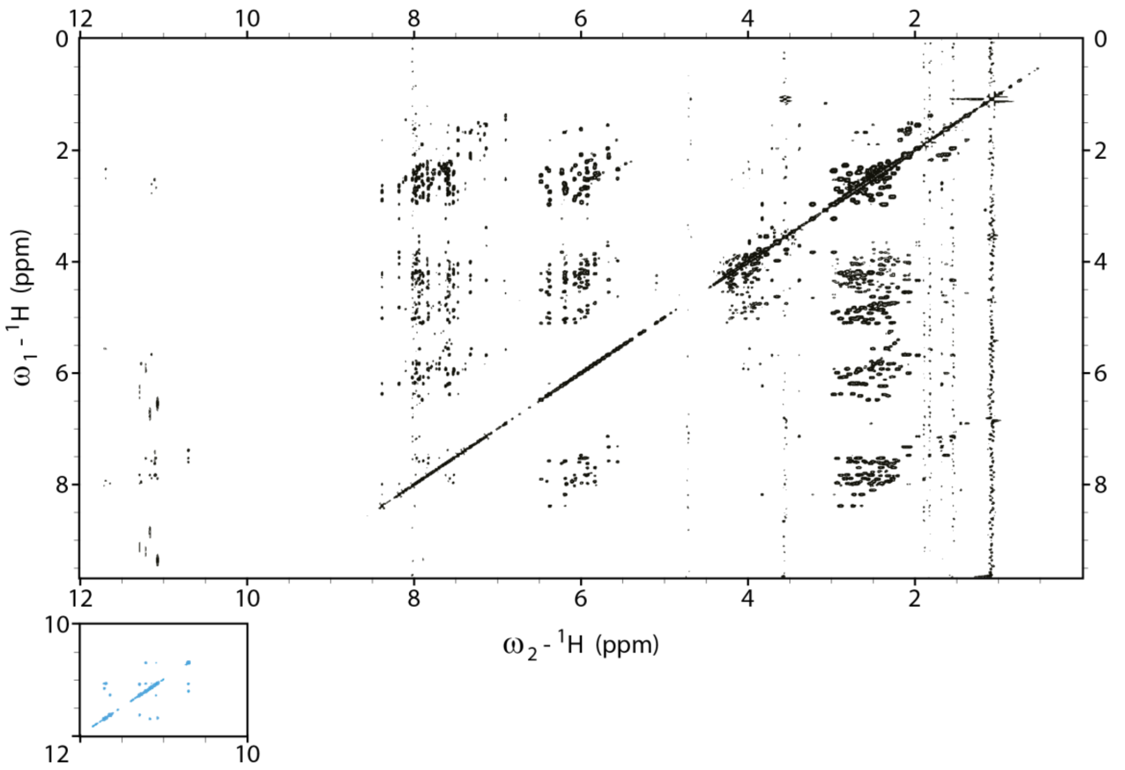

Supplementary Fig. 15 The full 2D-NOESY spectrum of Pu24m1 DNA in H_2O. The H1-H1 region (10-12 ppm) is inserted as the spectra collected using a fold-back strategy. Conditions: 1.5 mM Pu24m1 DNA, pH 7, 50 mM K^+, 25 °C.

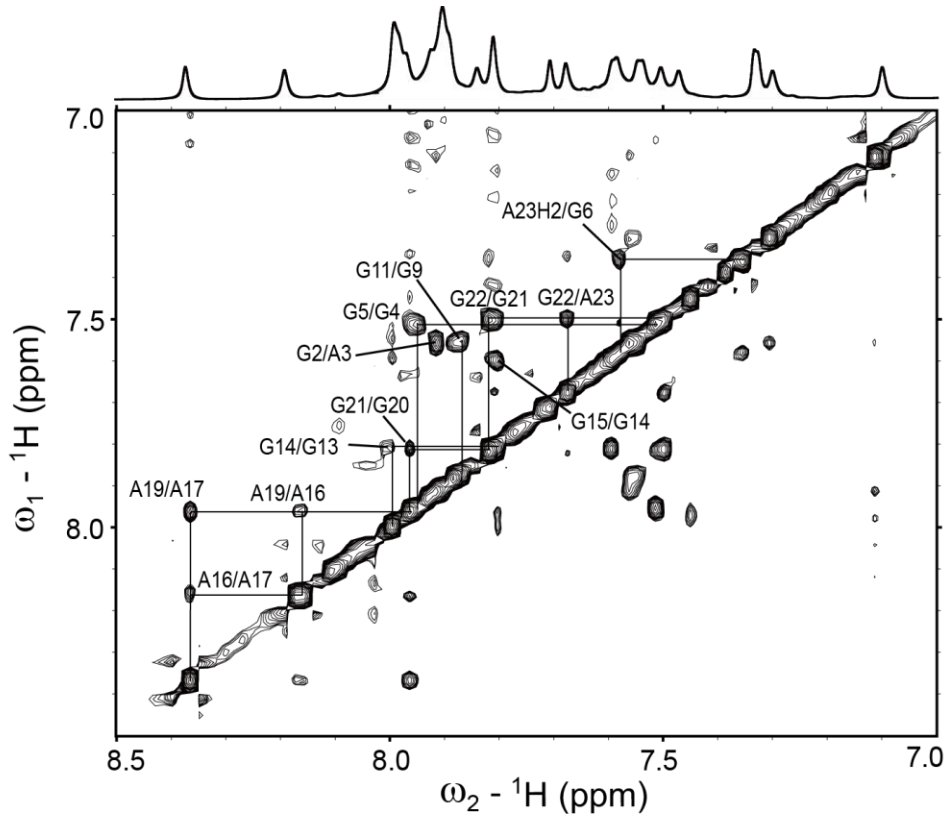

Supplementary Fig. 16 The H8−H8 region from the 2D-NOESY spectrum of Pu24m1 DNA in H_2O with sequential assignment pathway. Conditions: 1.5 mM Pu24m1 DNA, pH 7, 50 mM K^+, 25 °C.

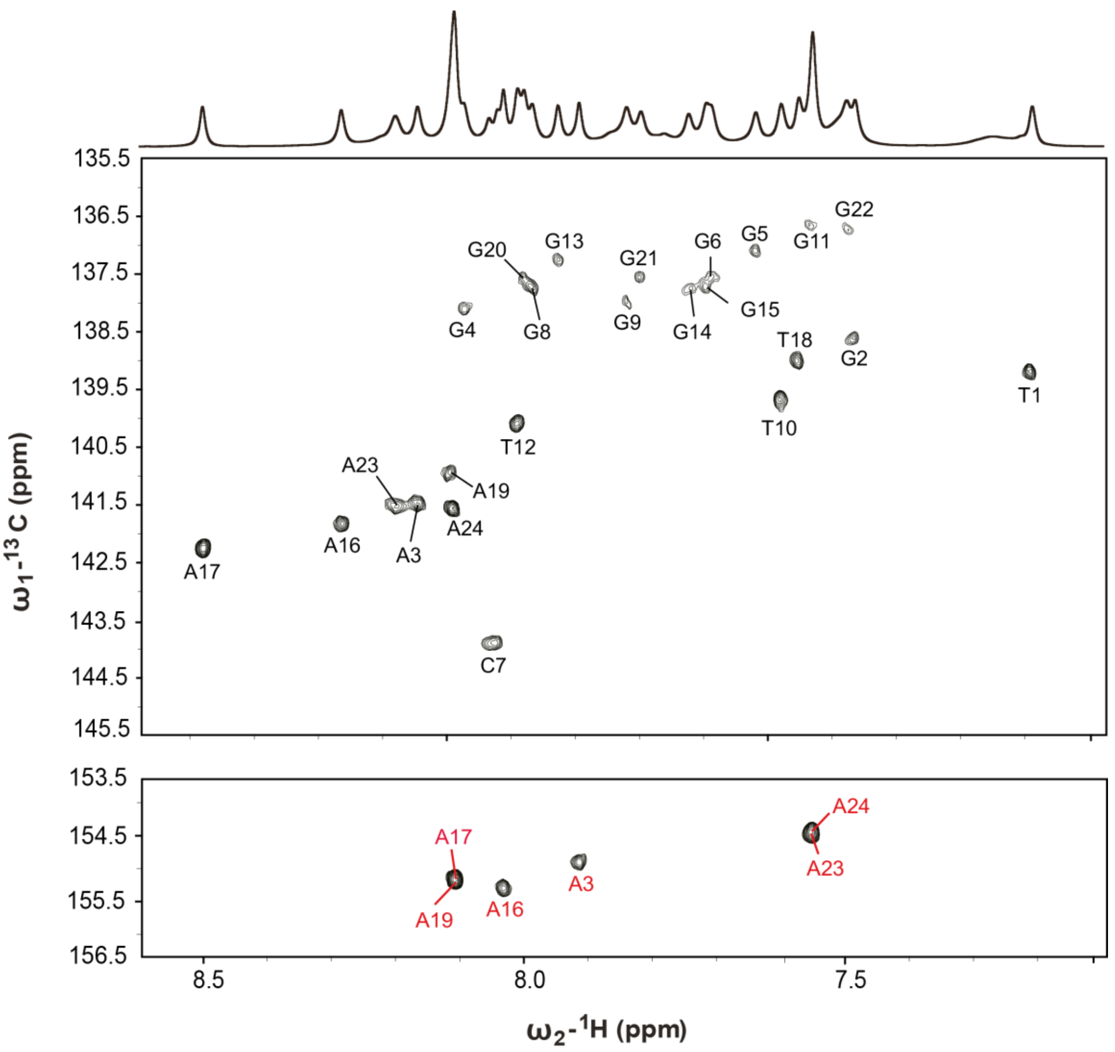

Supplementary Fig. 17 H6−C6/H8−C8 cross-peaks for all bases (black label) and H2-C2 contacts for adenine (red label) with assignments for BER-Pu24m1 complex by HSQC experiments. Conditions: 1.5 mM Pu24m1 DNA, BER: Pu24m1 = 3:1, pH 7, 50 mM K^+, 25 °C, DMSO-d_6 < 3.5%.

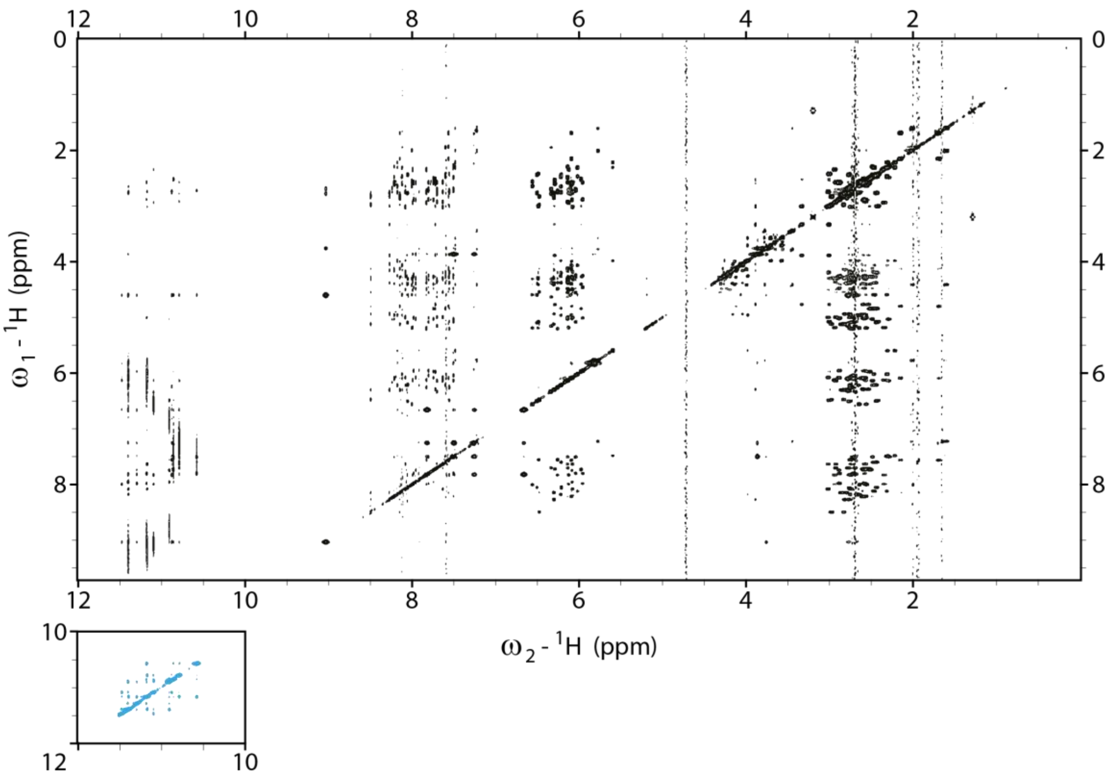

Supplementary Fig. 18 The full 2D-NOESY spectrum of BER-Pu24m1 complex in H_2O. The H1-H1 region (10-12 ppm) is inserted as the spectra collected using a fold-back strategy. Conditions: 1.5 mM Pu24m1 DNA, BER: Pu24m1 = 3:1, pH 7, 50 mM K^+, 35 °C, DMSO-d_6 < 3.5%.

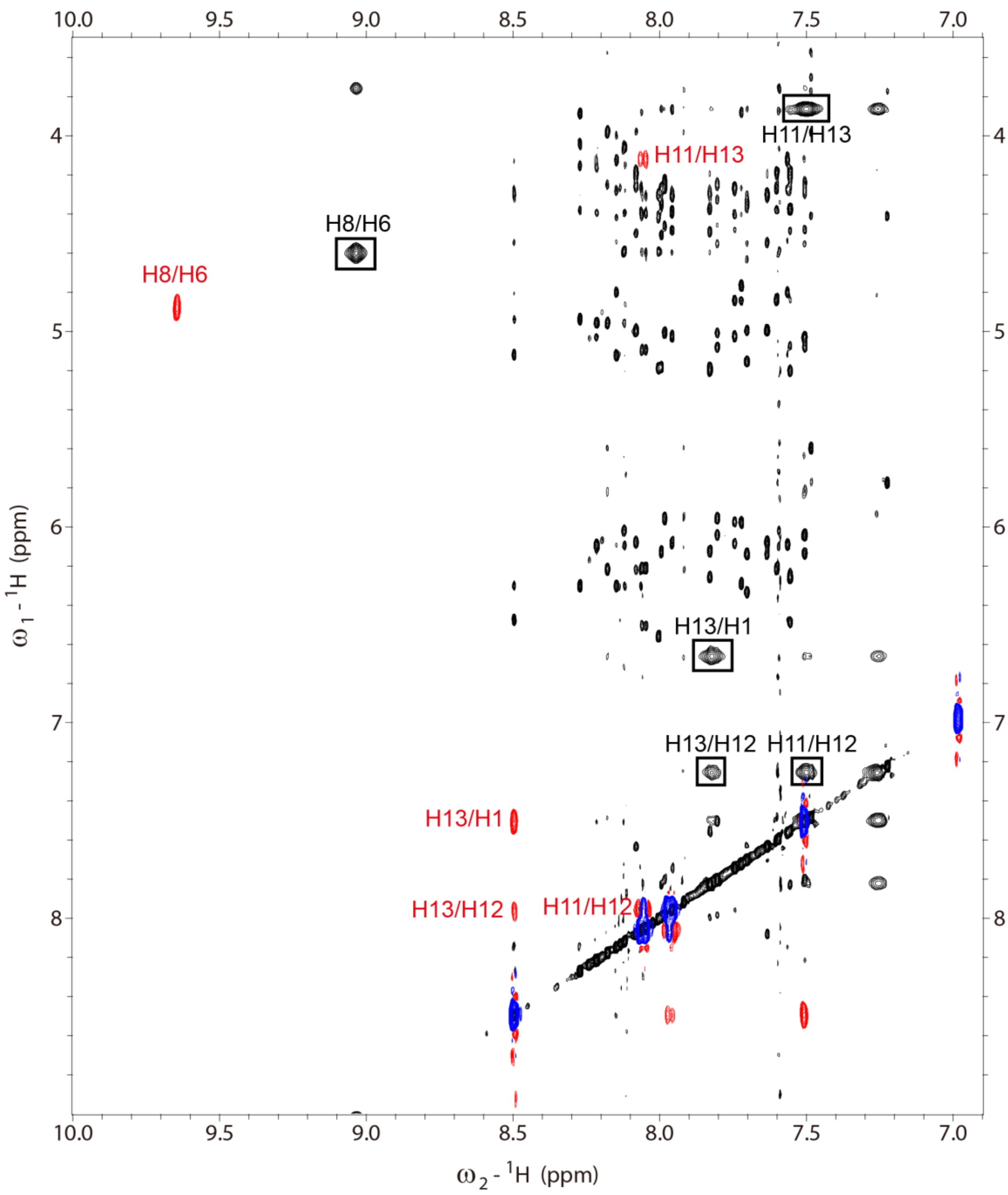

Supplementary Fig. 19 The 2D-NOESY spectra superposition of BER-Pu24m1 complex and free BER in H_2O, showing the differences in proton chemical shifts of BER in free and bound forms. The free and bound BER intramolecular NOEs are labeled in red and black (with boxes), respectively. Conditions: 1.5 mM Pu24m1 DNA, BER: Pu24m1 = 3:1, pH 7, 50 mM K^+, 35 °C, DMSO-d_6 < 3.5%.

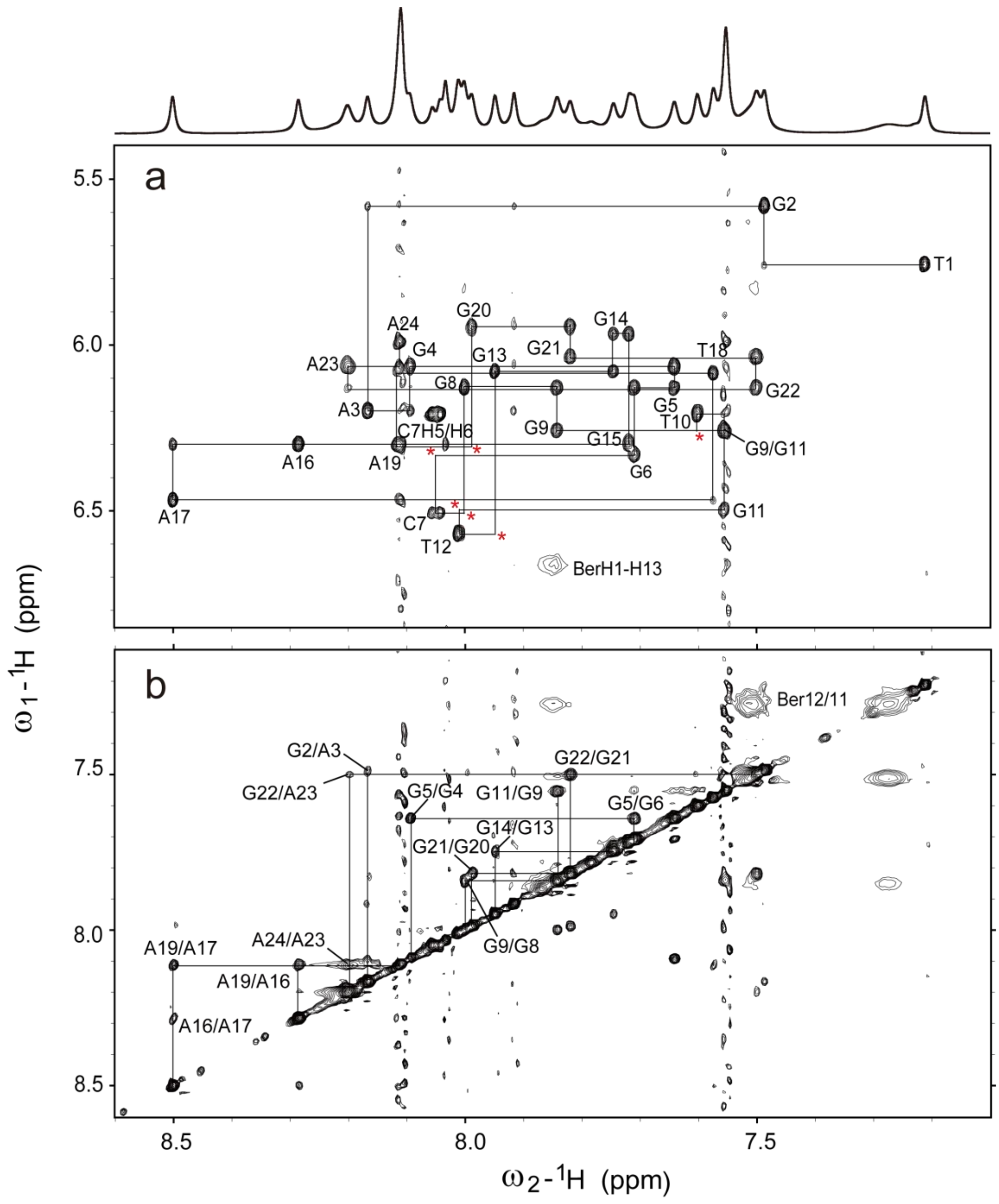

Supplementary Fig. 20 (a) The H1′-H6/H8 region and (b) H8−H8 region from the 2D-NOESY spectrum of BER-Pu24m1 complex in H_2O with sequential assignment pathway. Missing connectivity is labeled with red asterisks. Conditions: 1.5 mM Pu24m1 DNA, 3:1 BER/DNA, pH 7, 50 mM K^+, 25 °C, DMSO-d_6 < 3.5%.

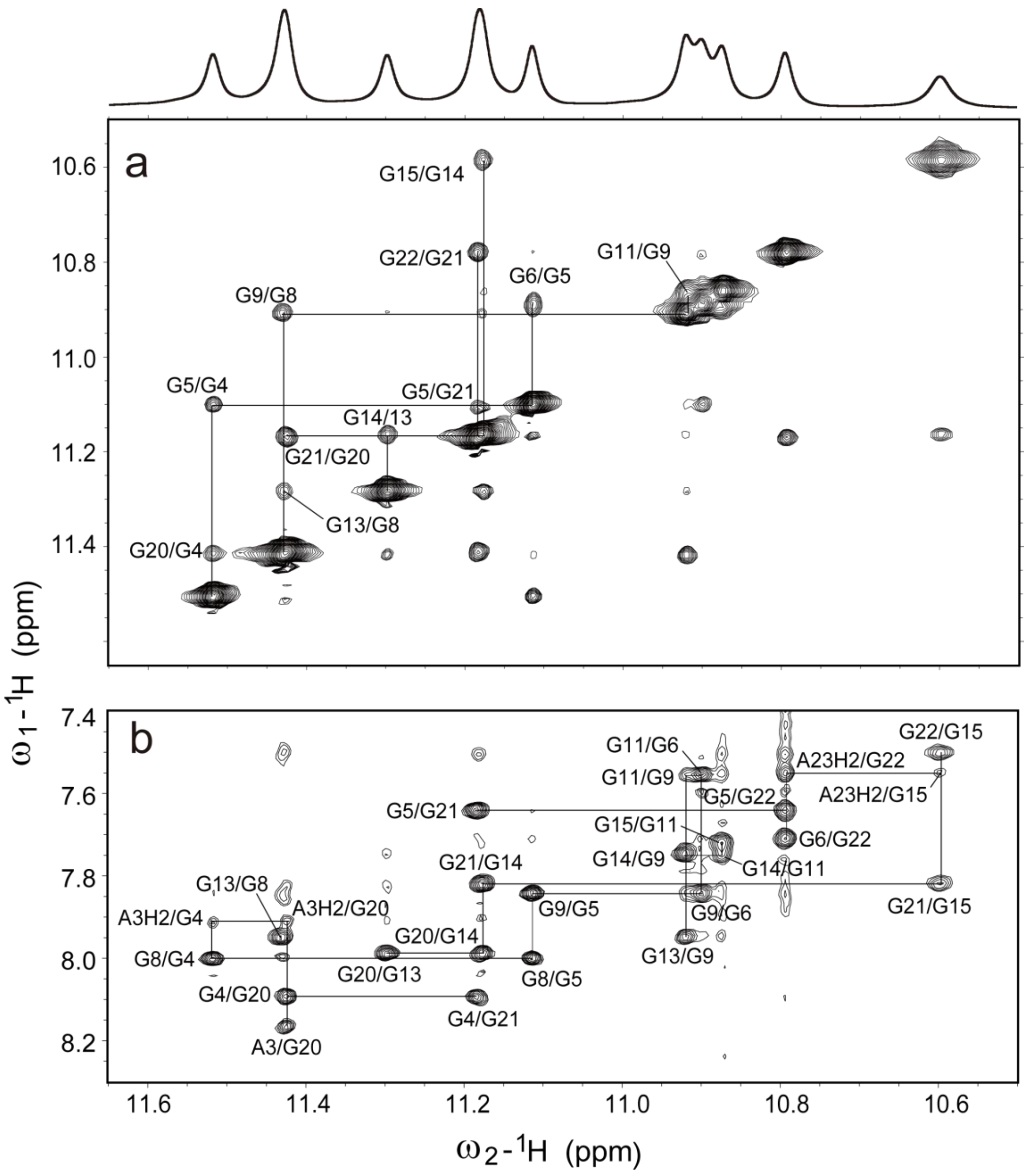

Supplementary Fig. 21 (a) The H1-H1 region and (b) H8−H1 region from the 2D-NOESY spectrum of BER-Pu24m1 complex in H_2O with sequential assignment pathway. Conditions: 1.5 mM Pu24m1 DNA, 3:1 BER/DNA, pH 7, 50 mM K^+, 25 °C, DMSO-d_6 < 3.5%.

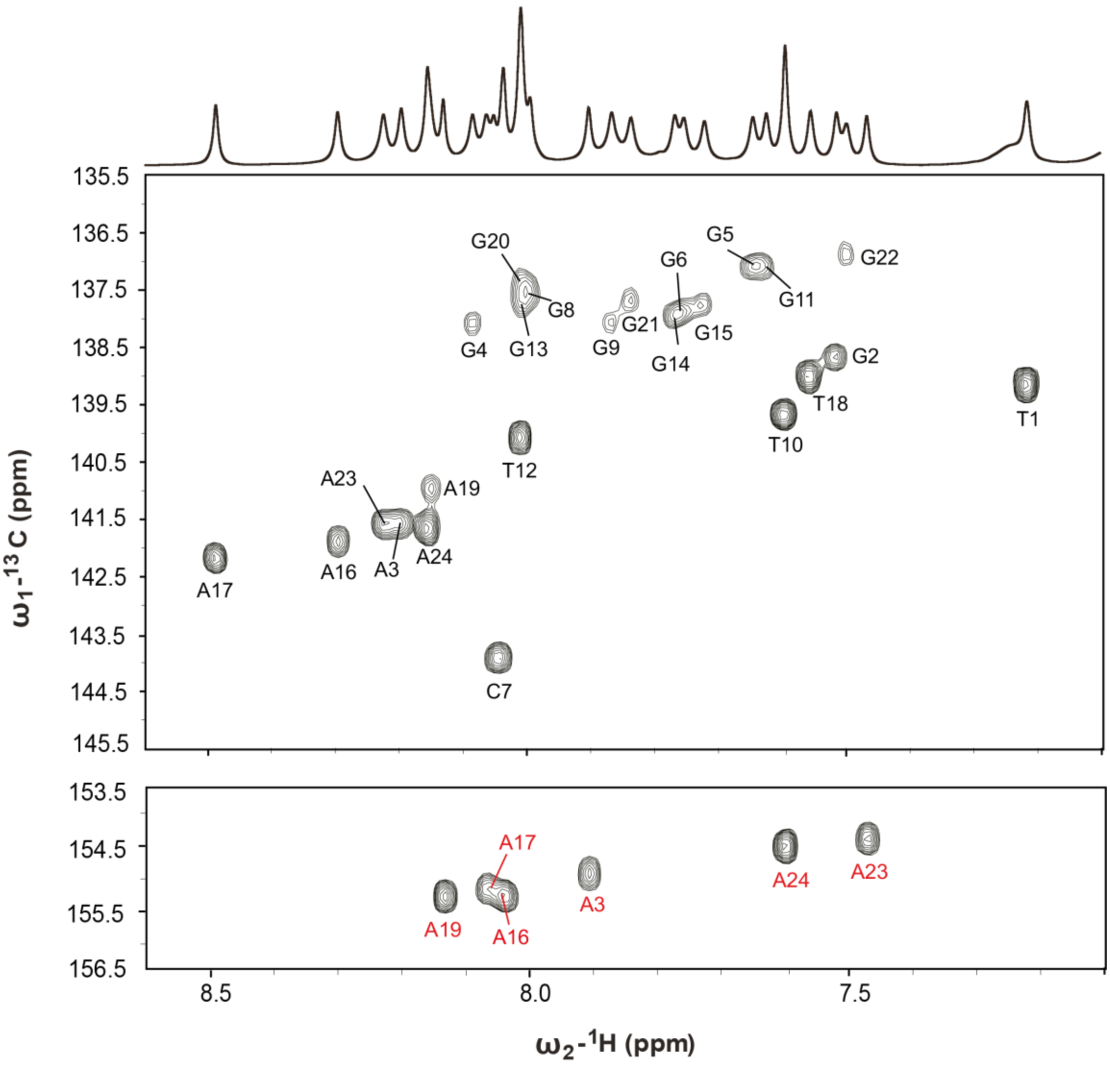

Supplementary Fig. 22 H6−C6/H8−C8 cross-peaks for all bases (black label) and H2-C2 contacts for adenines (red label) with assignments for COP-Pu24m1 complex by HSQC experiments. Conditions: 1.5 mM Pu24m1 DNA, COP:Pu24m1 = 2.5:1, pH 7, 50 mM K^+, 25 °C, DMSO-d_6 < 3.5%.

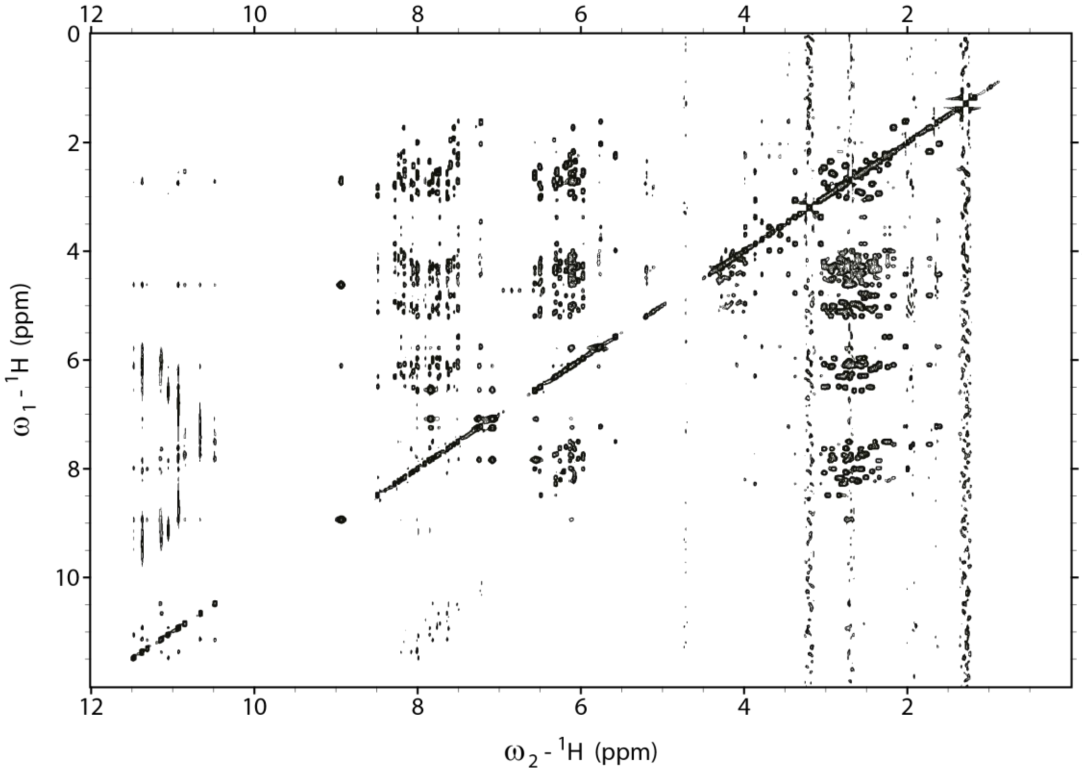

Supplementary Fig. 23 The full 2D-NOESY spectrum of COP-Pu24m1 complex in H_2O. Conditions: 1.5 mM Pu24m1 DNA, COP: Pu24m1 = 2.5:1, pH 7, 50 mM K^+, 35 °C, DMSO-d_6 < 3.5%.

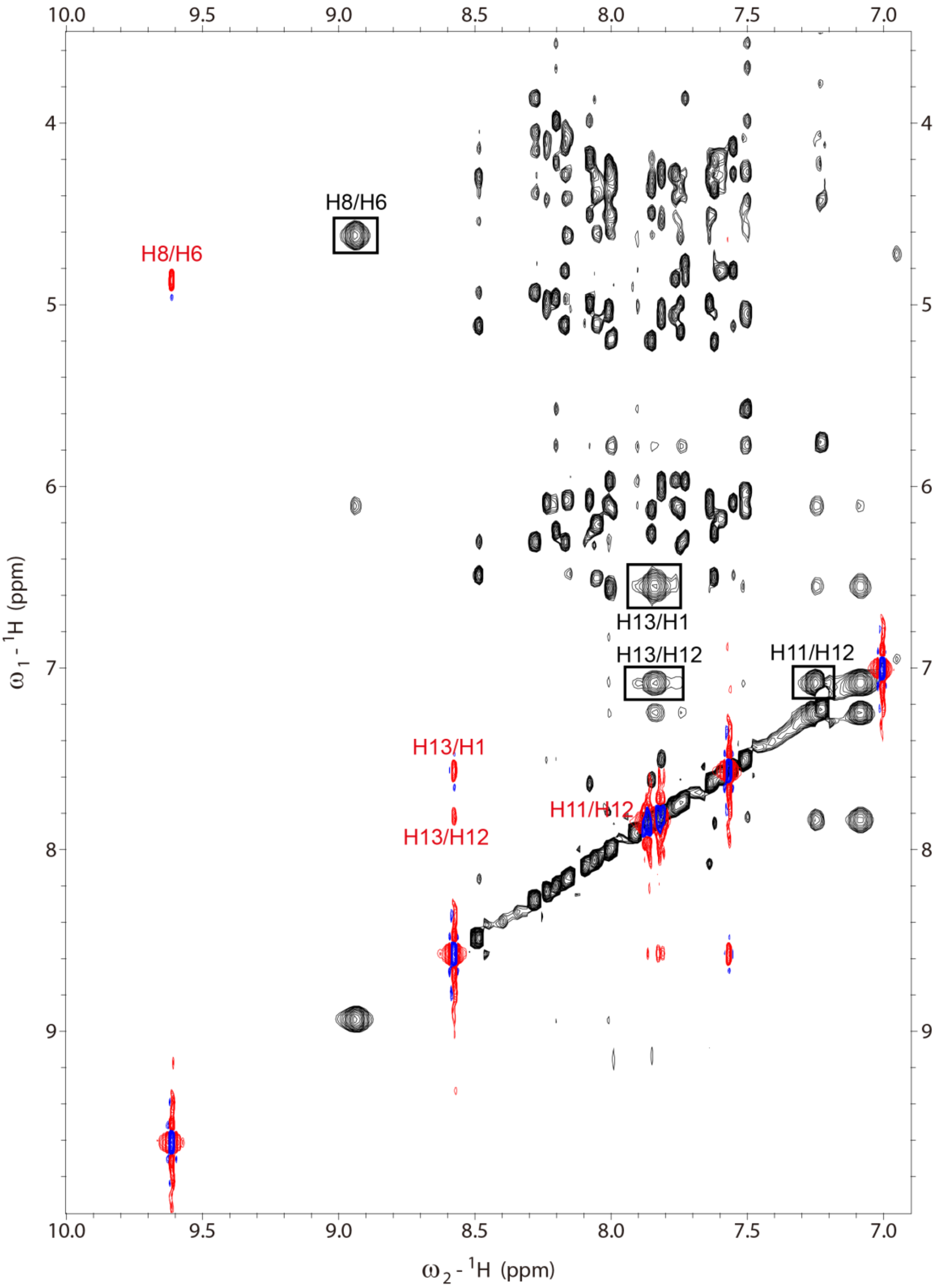

Supplementary Fig. 24 The 2D-NOESY spectra superposition of COP-Pu24m1 complex and free COP in H_2O, showing the differences in proton chemical shifts of COP in free and bound forms. The free and bound COP intramolecular NOEs are labeled in red and black (with boxes), respectively. Conditions: 1.5 mM Pu24m1 DNA, COP: Pu24m1 = 2.5:1, pH 7, 50 mM K^+, 35 °C, DMSO-d_6 < 3.5%.

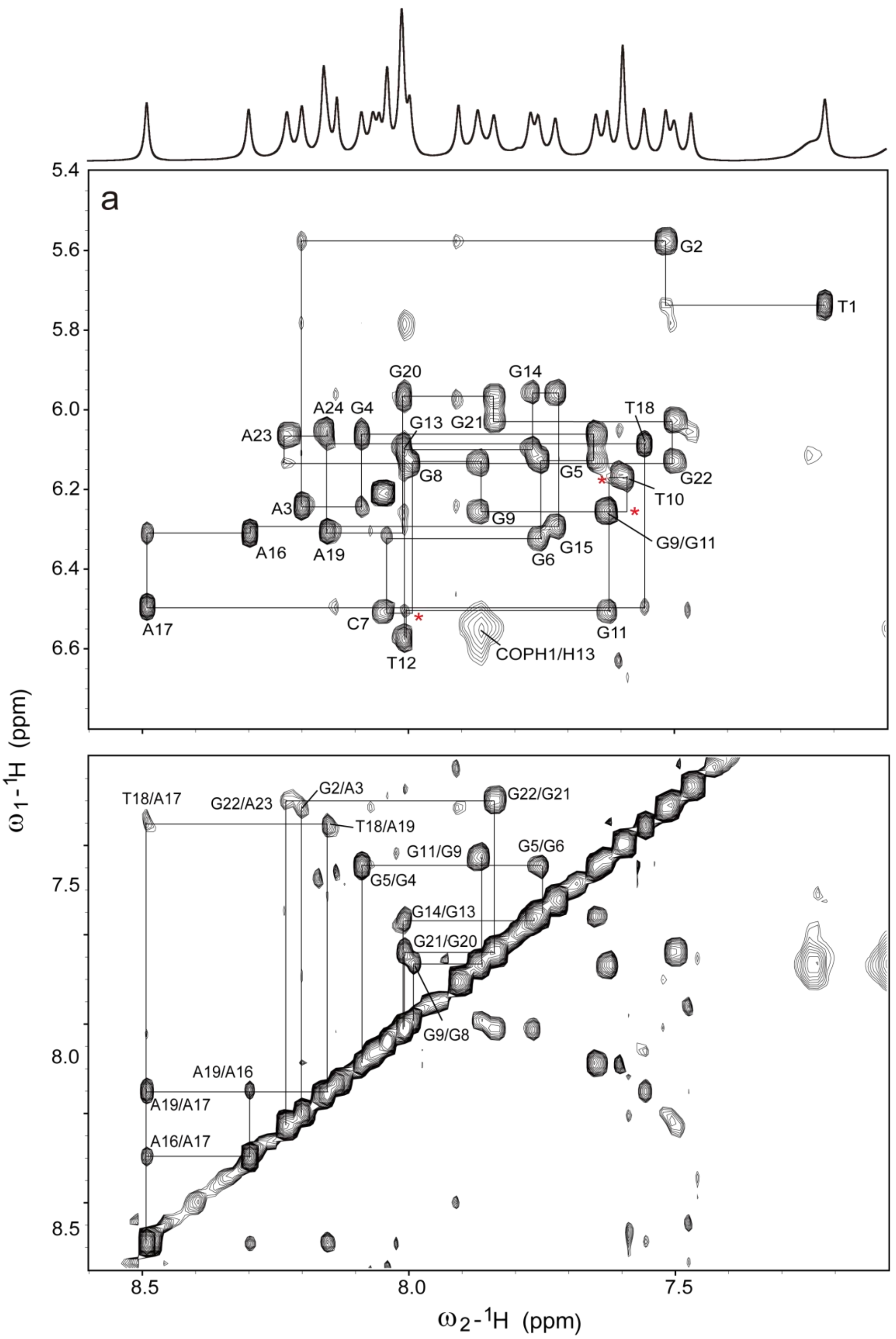

Supplementary Fig. 25 (a) The H1′-H6/H8 region and (b) the H8−H8 region from the 2D-NOESY spectrum of COP-Pu24m1 complex in H_2O with sequential assignment pathway. Missing connectivity is labeled with red asterisks. Conditions: 1.5 mM Pu24m1 DNA, COP: Pu24m1 = 2.5:1, pH 7, 50 mM K^+, 25 °C, DMSO-d_6 < 3.5%.

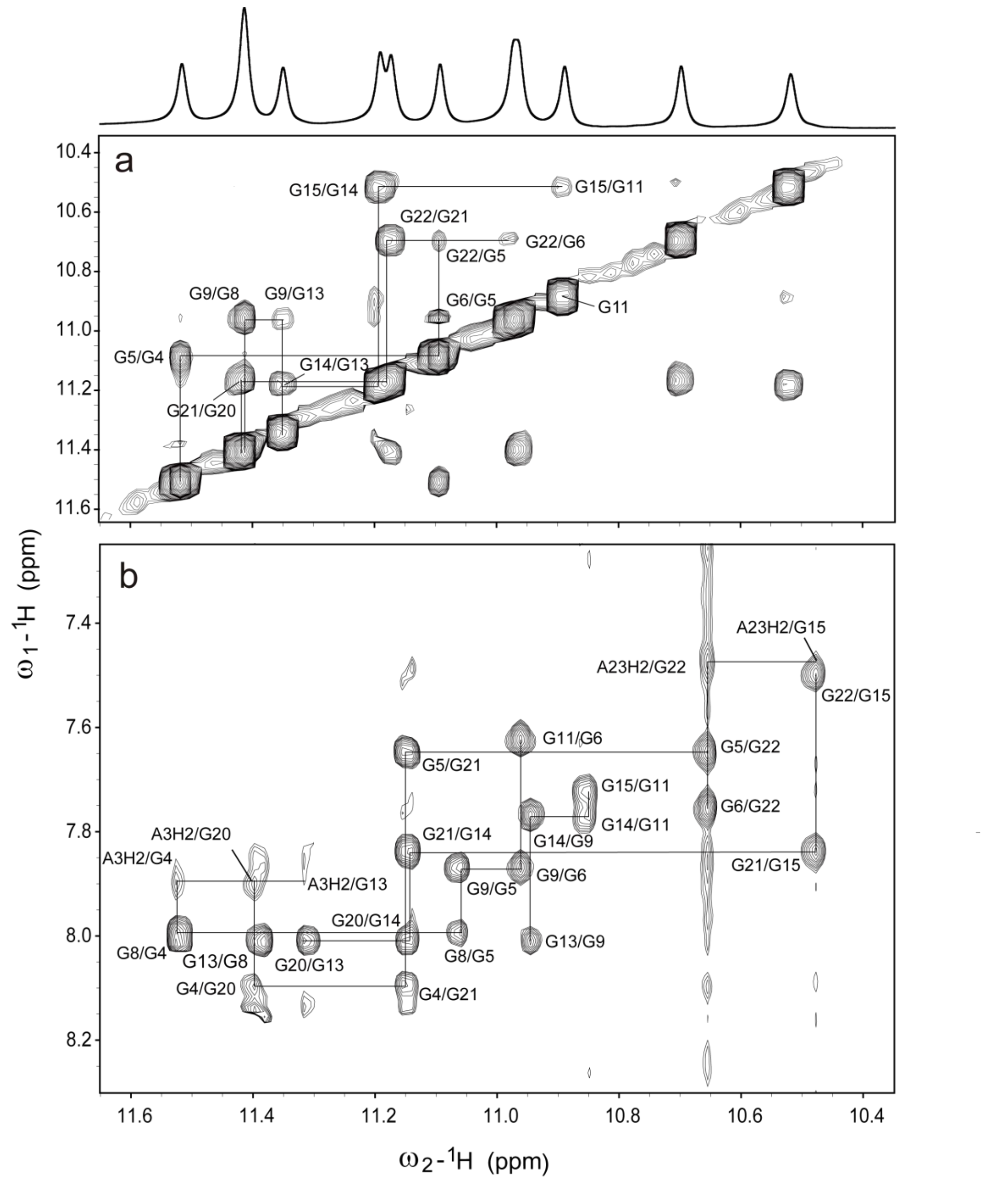

Supplementary Fig. 26 (a) The H1-H1 region and (b) the H8−H1 region from the 2D-NOESY spectrum of COP-Pu24m1 complex in H_2O with sequential assignment pathway. Conditions: 1.5 mM Pu24m1 DNA, COP: Pu24m1 = 2.5:1, pH 7, 50 mM K^+, 25 °C, DMSO-d_6 < 3.5%.

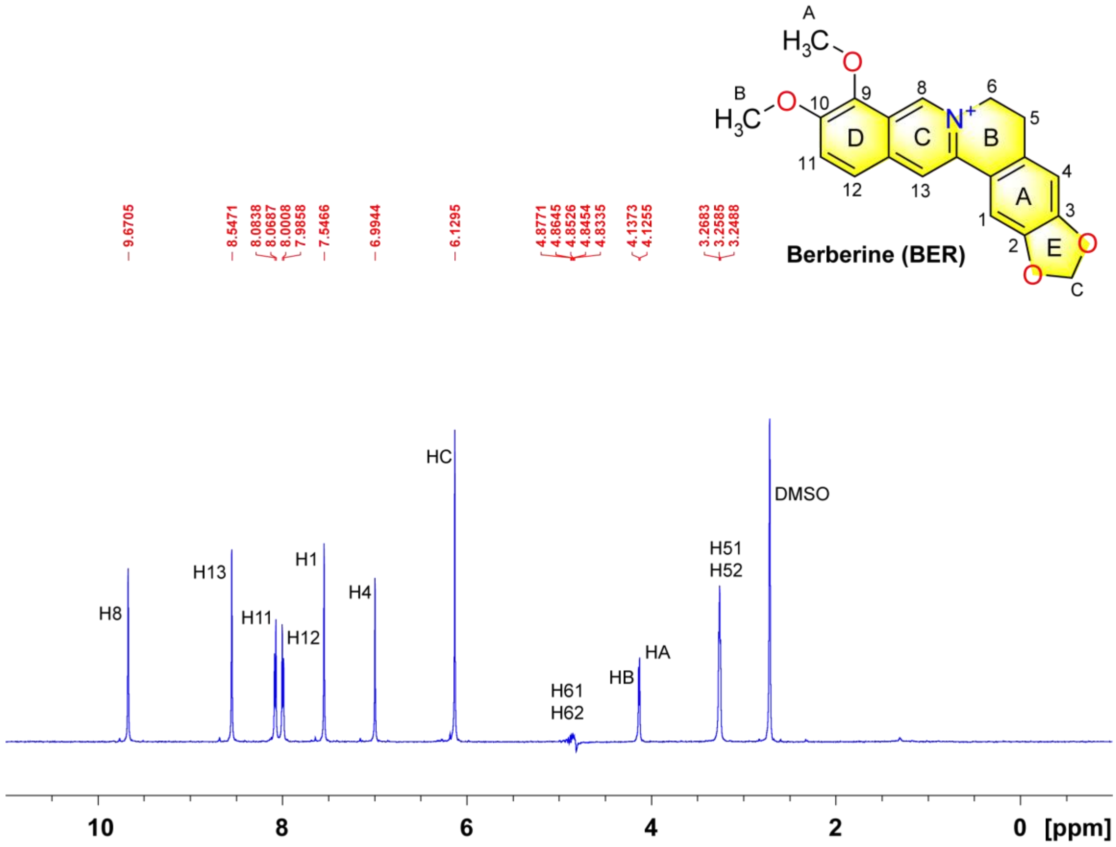

Supplementary Fig. 27 1D ^{1}H NMR spectrum of free berberine in pH 7, 50 mM K^{+}-containing solution at 25 °C with peak assignments and corresponding chemical structure, DMSO-d_6 < 1.5%.

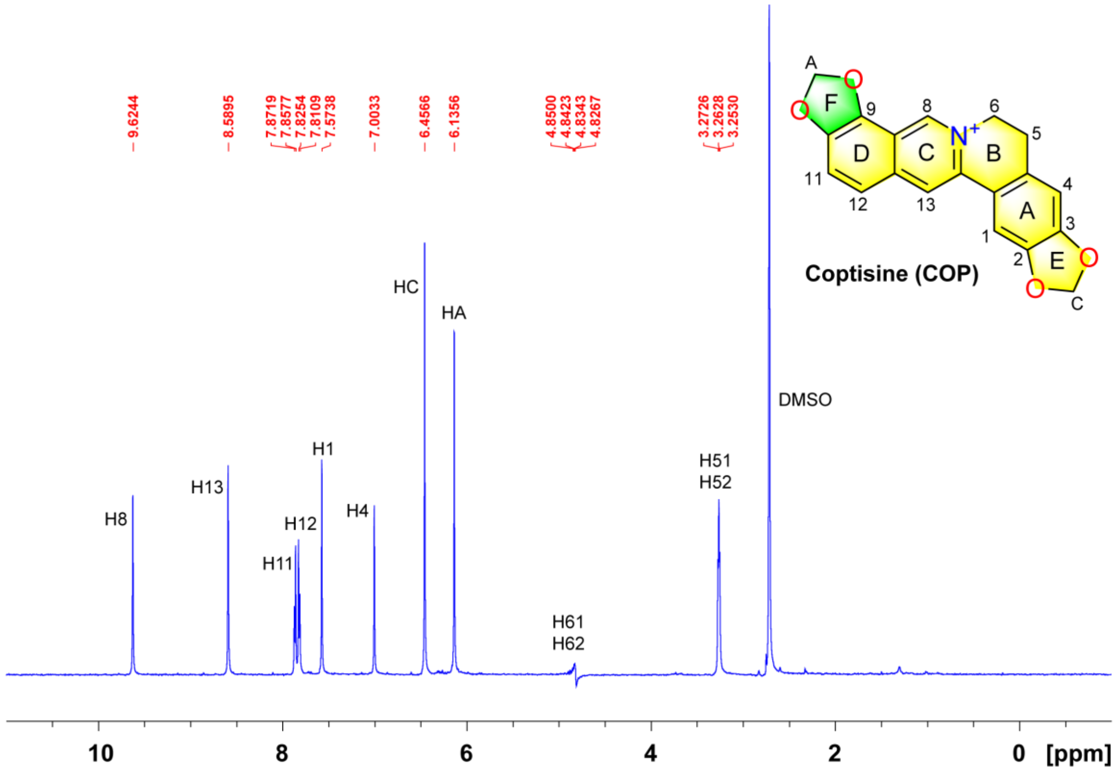

Supplementary Fig. 28 1D ^{1}H NMR spectrum of free coptisine in pH 7, 50 mM K^+-containing solution at 25 °C with peak assignments and corresponding chemical structure, DMSO-d_6 < 3.5%.

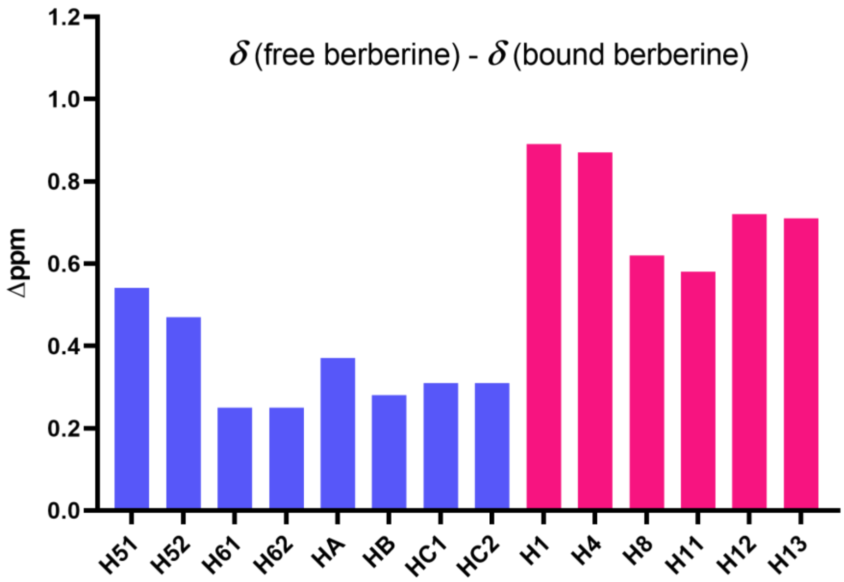

Supplementary Fig. 29 The proton chemical shift differences between the free-berberine and bound-berberine at 25 °C. The proton numbering of berberine is shown. The chemical shift differences of the aromatic and nonaromatic protons are colored by red and blue, respectively.

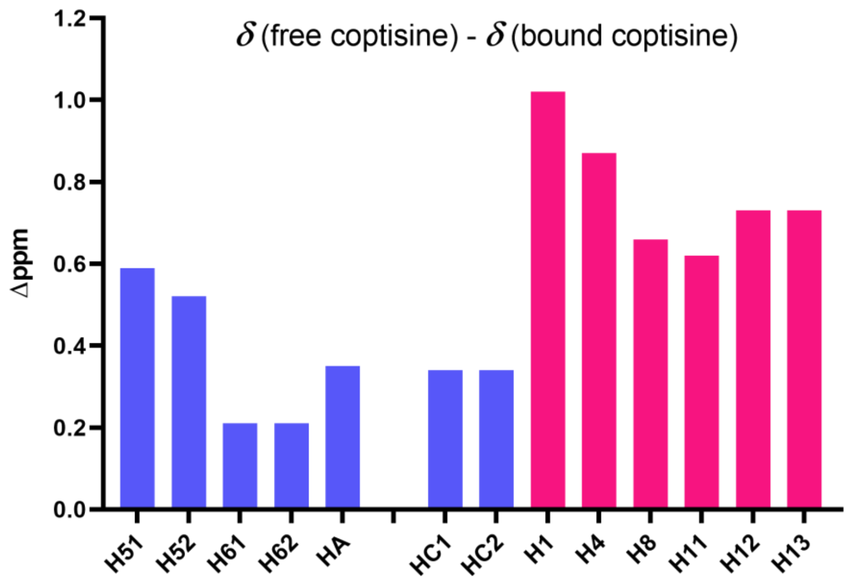

Supplementary Fig. 30 The proton chemical shift differences between the free-coptisine and bound-coptisine at 25 °C. The proton numbering of coptisine is shown. The chemical shift differences of the aromatic and nonaromatic protons are colored by red and blue, respectively.

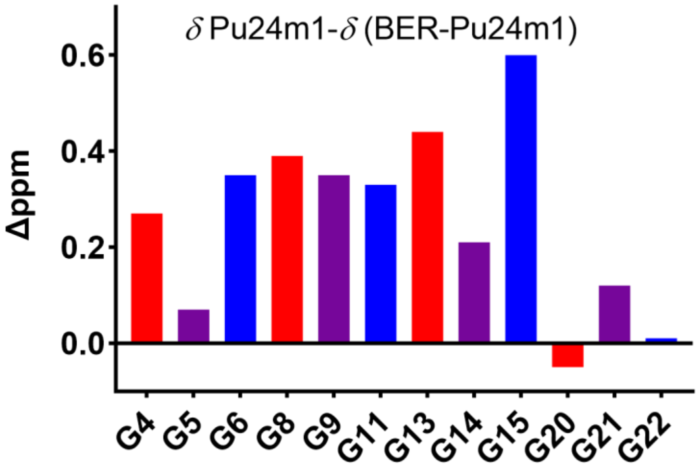

Supplementary Fig. 31 The H1 proton chemical shift differences between the free Pu24m1 DNA and its complex with BER at 25 °C. The residue numbers of Pu24m1 DNA are shown.

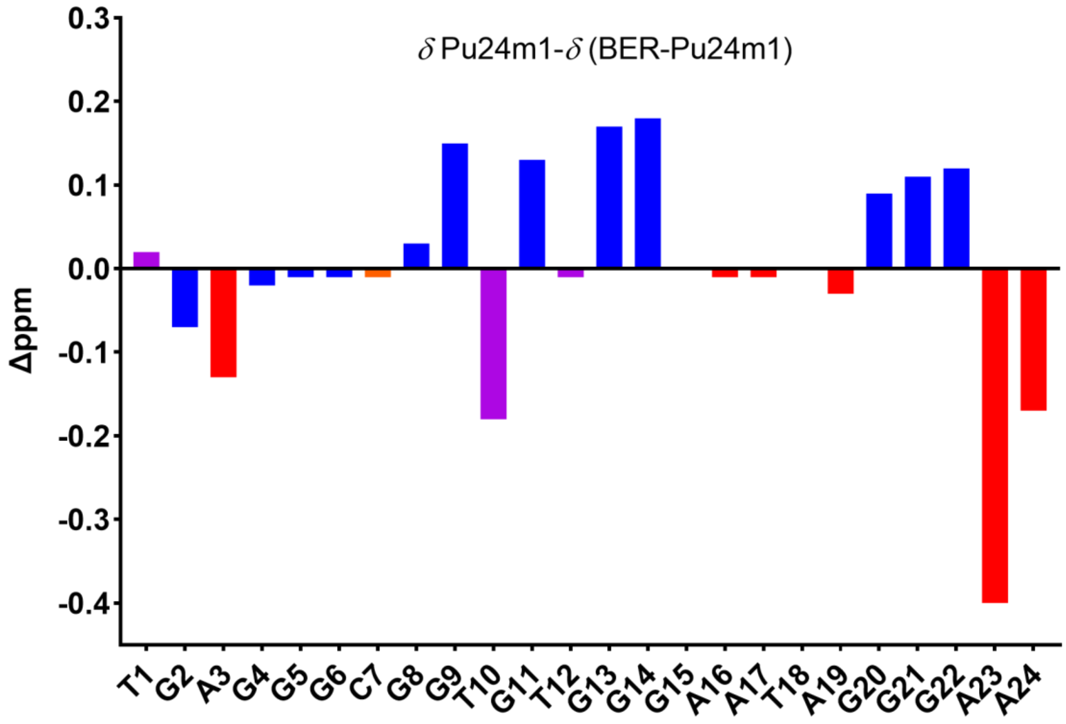

Supplementary Fig. 32 The H8/H6 proton chemical shift differences between the free Pu24m1 DNA and its complex with BER at 25 °C. The residue numbers of Pu24m1 DNA are shown.

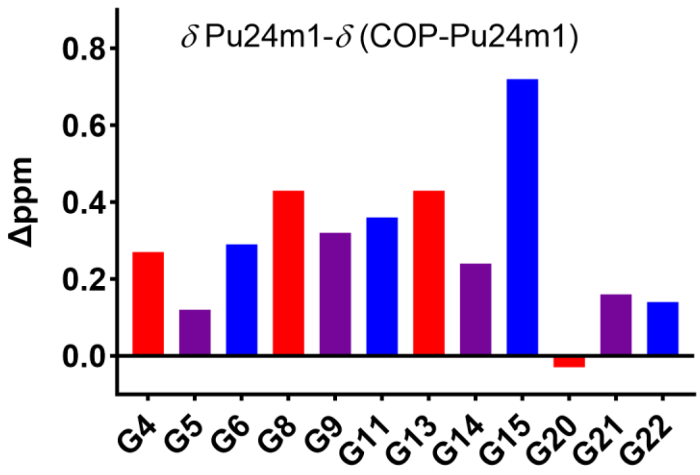

Supplementary Fig. 33 The H1 proton chemical shift differences between the free Pu24m1 DNA and its complex with COP at 25 °C. The residue numbers of Pu24m1 DNA are shown.

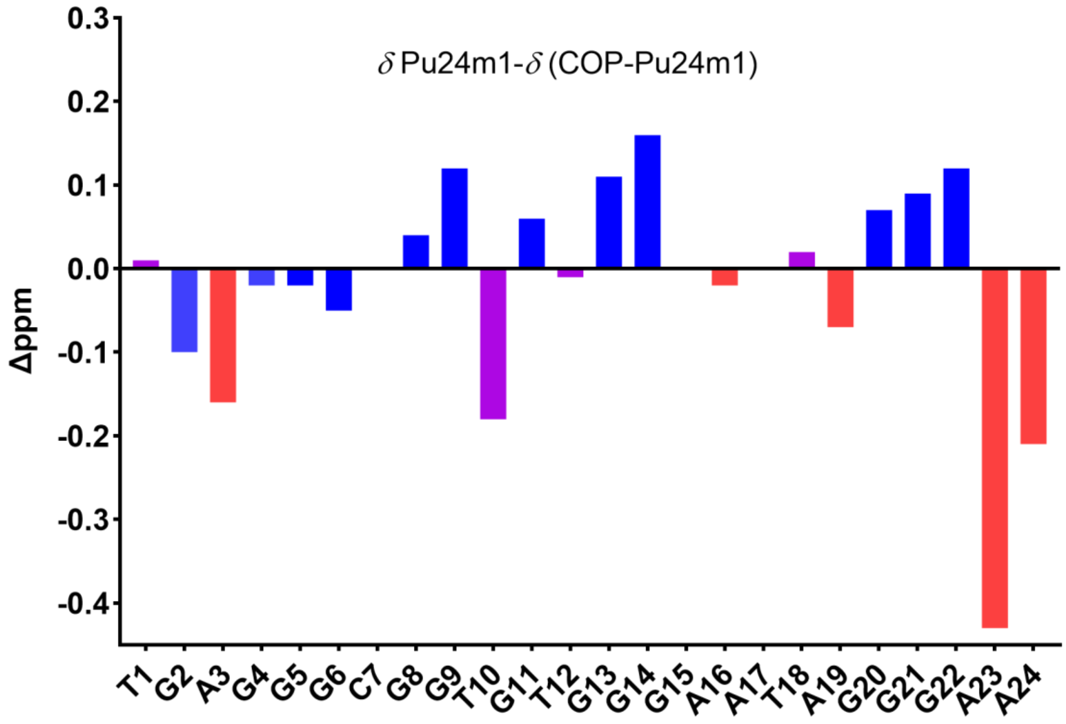

Supplementary Fig. 34 The H8/H6 proton chemical shift differences between the free Pu24m1 DNA and its complex with COP at 25 °C. The residue numbers of Pu24m1 DNA are shown.

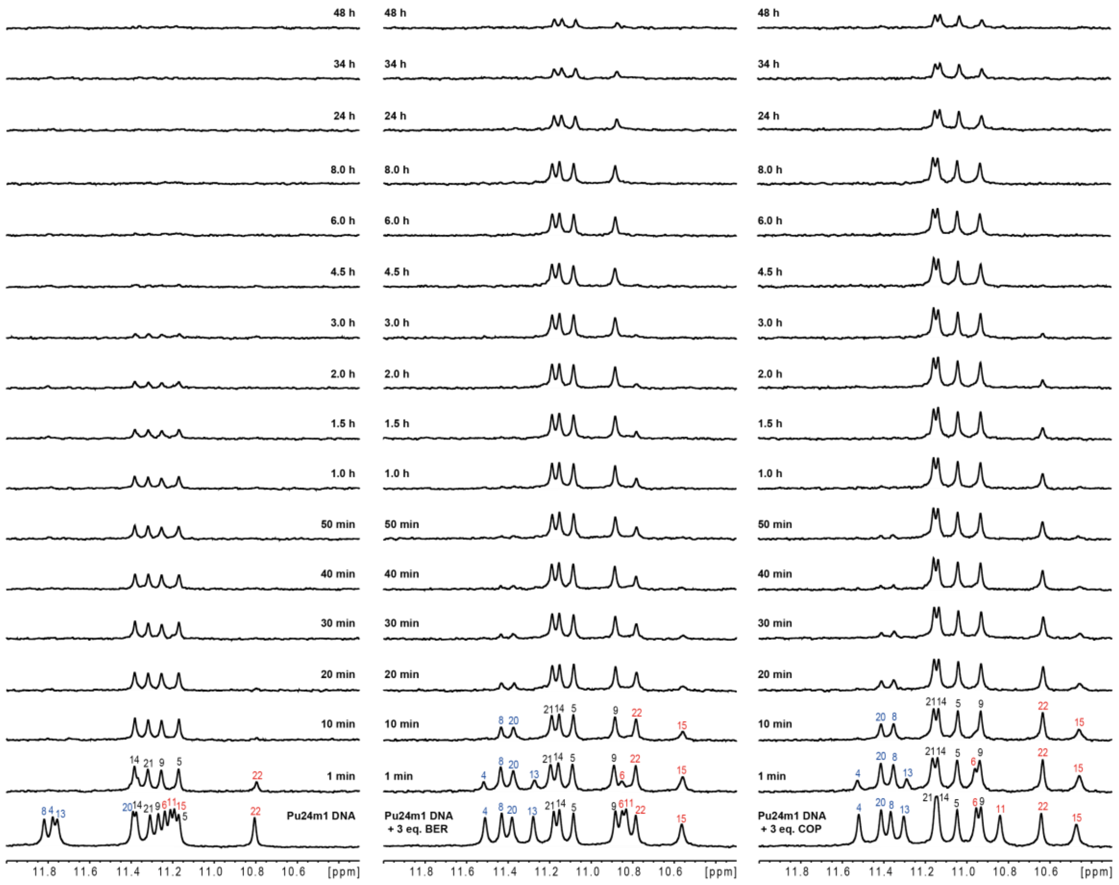

Supplementary Fig. 35 The solvent exchange experiments for Pu24m1 DNA and its complex with berberine (BER) and coptisine (COP), respectively. Imino proton spectra of Pu24m1 DNAs in H_2O (bottom) and after dissolving the samples in D_2O at different times were shown. Conditions: 150 μM DNA, Ligand: Pu24m1 = 3:1, pH 7, 50 mM K^+ solution, 25 °C.

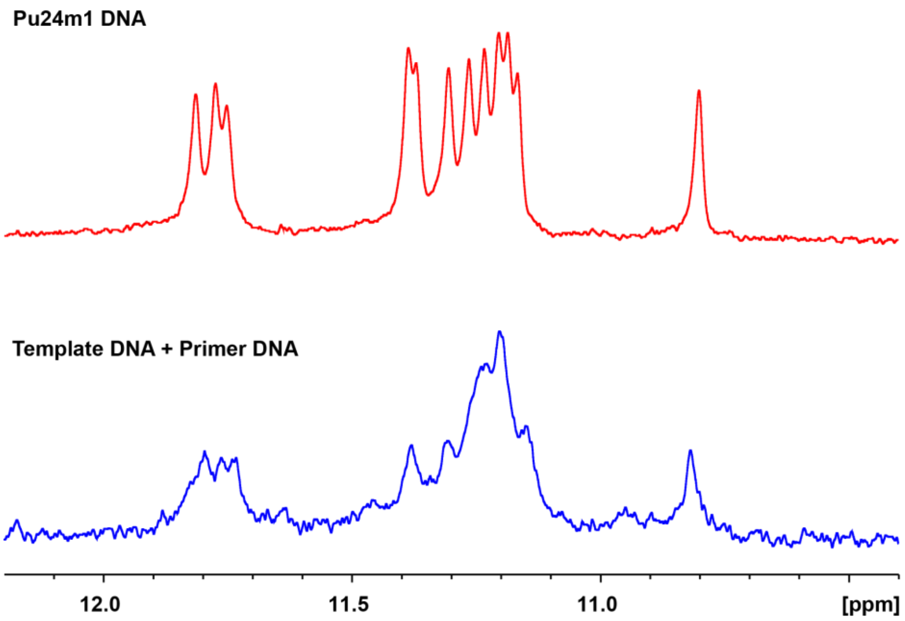

Supplementary Fig. 36 NMR imino proton region of Pu24m1 DNA and *KRAS* template DNA mixed with its primer DNA, respectively. Conditions: 150 μM DNA, pH 7, 100 mM K^+ solution, 25 °C.

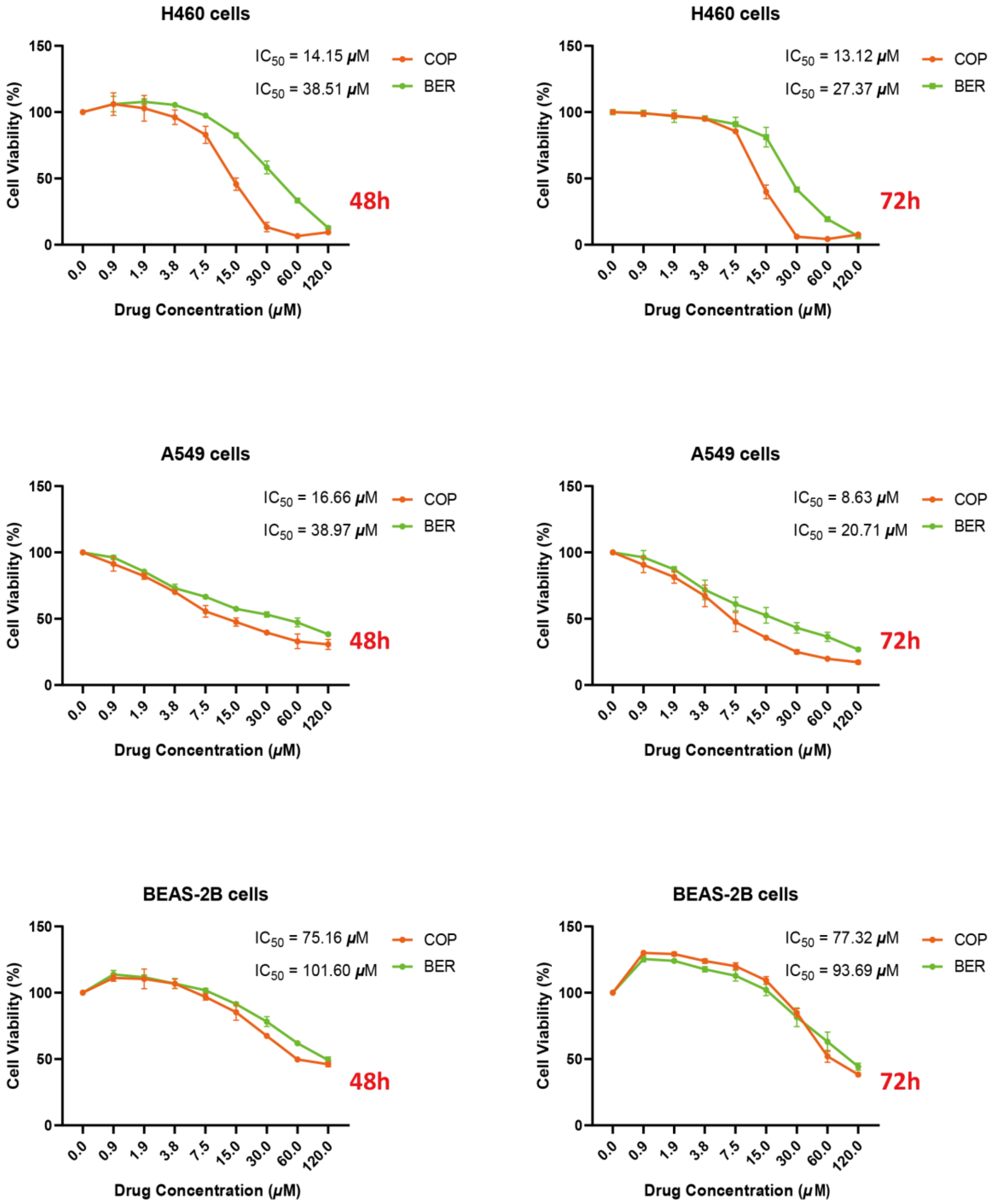

Supplementary Fig. 37 The dose-response curves of H460, A549, and BEAS-2B cells with the treatment of berberine and coptisine for 48 and 72 hours, respectively. The determined IC_{50} values are shown. The experiments were run in triplicate. Data are presented as mean values ± SD.

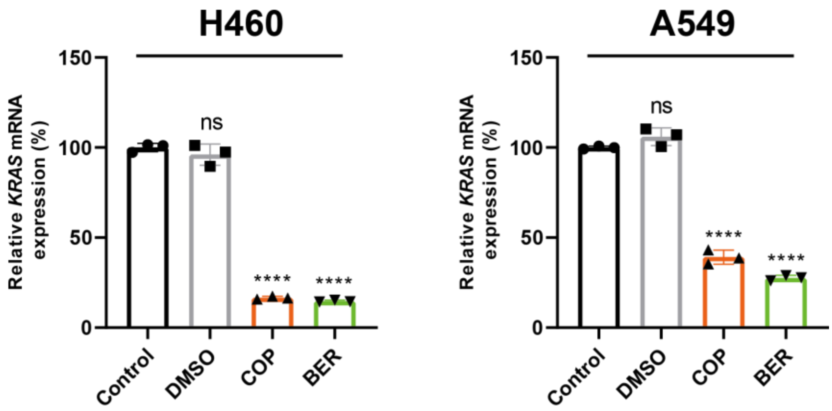

Supplementary Fig. 38 qRT-PCR results show that berberine (24 μM) and coptisine (10 μM) significantly lower the *KRAS* mRNA levels in H460 and A549 cancer cells for 48 h, while not the DMSO (<1%). H_2O was used as the negative control (no inhibition, 100%). The relative *KRAS* mRNA levels were normalized with *GAPDH*. The experiments were run in triplicate. Data are presented as mean values ± SD. *P* values (ns, P = 0.3661 for H460 and P = 0.1277 for A549; ****, P < 0.0001) were determined by one-way ANOVA with post hoc Dunnett, relative to H_2O control.

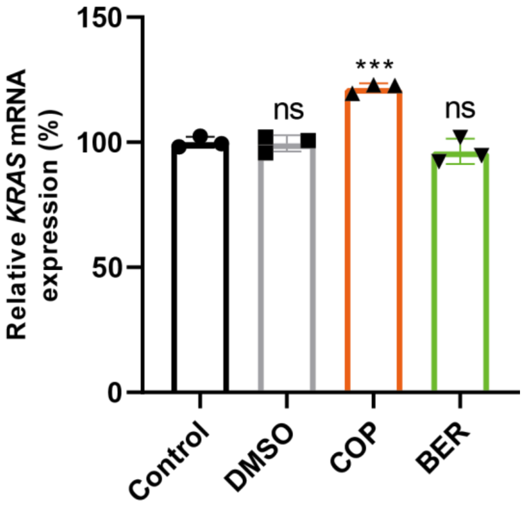

Supplementary Fig. 39 qRT-PCR results show that DMSO (<1%), berberine (24 μM), and coptisine (10 μM) do not lower the *KRAS* mRNA levels in normal human bronchial epithelial cells (BEAS-2B) for 24 h. H_2O was used as the negative control (no inhibition, 100%). The relative *KRAS* mRNA levels were normalized with *GAPDH*. The experiments were run in triplicate. Data are presented as mean values ± SD. *P* values (ns, P = 0.9962 for DMSO and P = 0.4386 for BER; ***, P < 0.001) were determined by one-way ANOVA with post hoc Dunnett, relative to H_2O control.

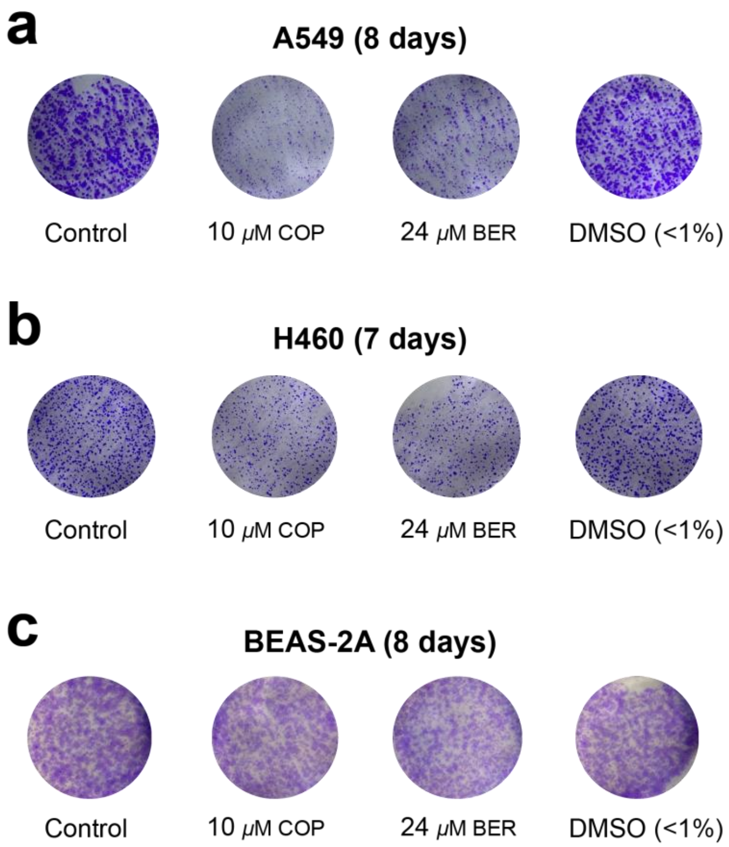

Supplementary Fig. 40 Colony formation of (a) A549, (b) H460, and (c) BEAS-2A cells in the presence and absence of COP, BER, and DMSO, respectively, for 7 or 8 days. The experiments were run in duplicate.

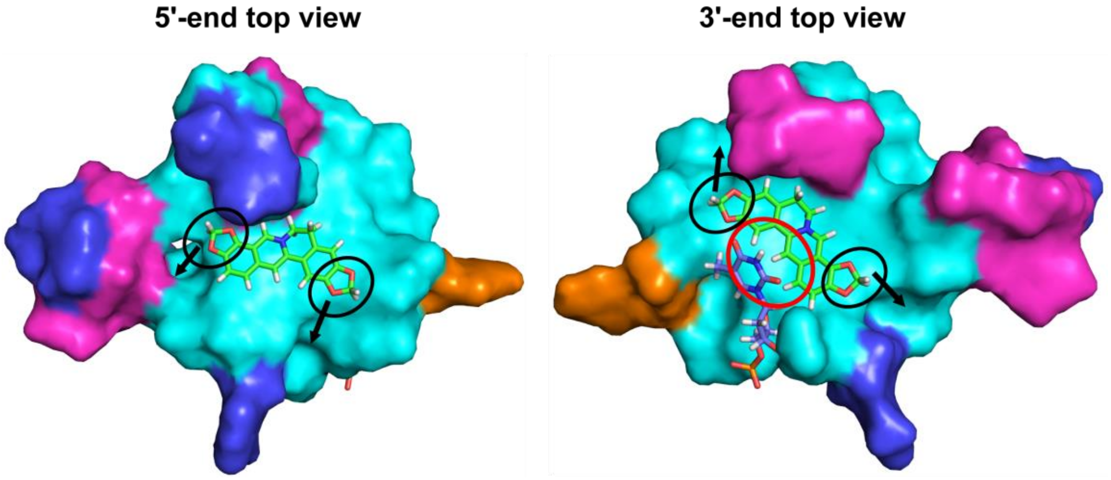

Supplementary Fig. 41 Suggested modifications of coptisine to enable additional interaction with the *KRAS*-G4. The black circles indicate the positions to be modified by introducing side chains for groove interactions. The black arrows show the grooves at 5′- and 3′-sites where the attached side chain will locate in. The red circle suggests the positions to introduce hydrogen bonds between coptisine and thymine T10. The *KRAS*-G4 is shown in cartoon representation. Cyan, guanine; magenta, adenine; blue, thymine; orange, cytosine; green, coptisine.

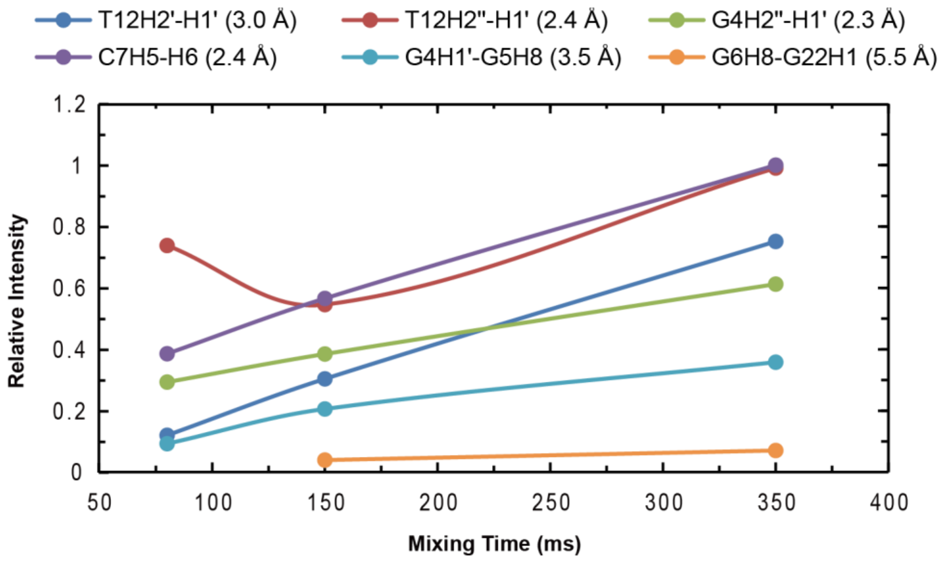

Supplementary Fig. 42 Representative NOE build-up curves. The NOE intensities were obtained from a series of 2D NOESY experiments and normalized by the intensity of cytosine H5/H6 NOE at 350 ms. The distances and assignments between the interacting spin pairs are indicated. Conditions: 1.5 mM Pu24m1 DNA, pH 7, 50 mM K^+, 25 °C.

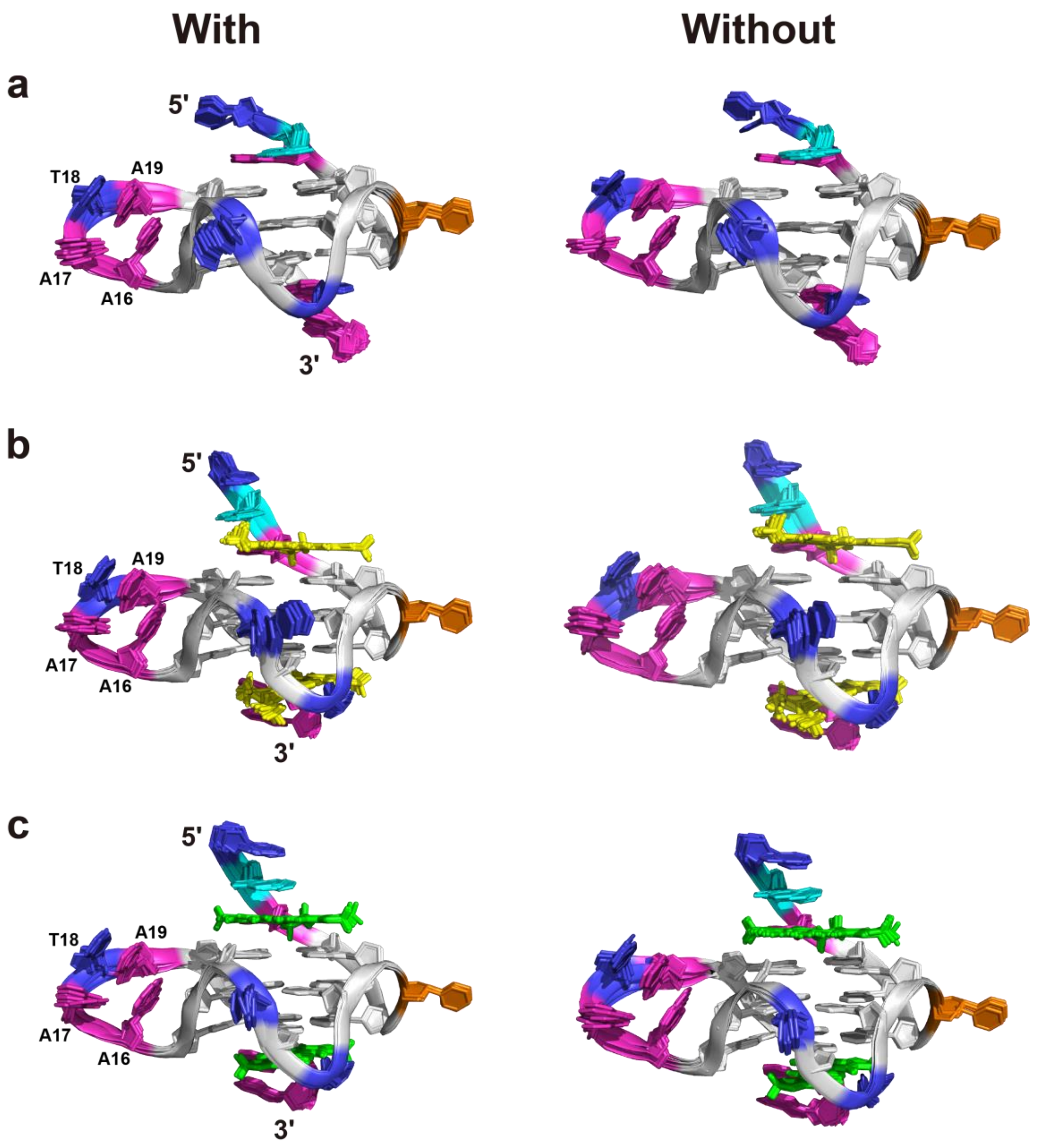

Supplementary Fig. 43 Superposition of the 10 lowest energy NMR structures of the free *KRAS*-G4 (a), berberine-*KRAS*-G4 (b), and coptisine-*KRAS*-G4 (c) with G-tetrad planarity restraints (left) and without G-tetrad planarity restraints (right). Yellow, berberine; green, coptisine; cyan, flanking guanine; gray, tetrad guanine; magenta, adenine; blue, thymine; orange, cytosine.
